# Comparative transcriptomes and WGCNA reveal hub genes for spike germination in different quinoa lines

**DOI:** 10.1186/s12864-024-11151-y

**Published:** 2024-12-20

**Authors:** Liubin Huang, Lingyuan Zhang, Ping Zhang, Junna Liu, Li Li, Hanxue Li, Xuqin Wang, Yutao Bai, Guofei Jiang, Peng Qin

**Affiliations:** Yunnan Agricultural University, Kunming, China

**Keywords:** WGCNA, Quinoa, Spike germination, Physiological properties, Metabolites, Transcription factors

## Abstract

**Background:**

Quinoa, as a new food crop, has attracted extensive attention at home and abroad. However, the natural disaster of spike germination seriously threatens the quality and yield of quinoa. Currently, there are limited reports on the molecular mechanisms associated with spike germination in quinoa.

**Results:**

In this study, we utilized transcriptome sequencing technology and successfully obtained 154.51 Gb of high-quality data with a comparison efficiency of more than 88%, which fully demonstrates the extremely high reliability of the sequencing results and lays a solid foundation for subsequent analysis. Using these data, we constructed a weighted gene co-expression network (WGCNA) related to starch, sucrose, α-amylase, and phenolic acid metabolites, and screened six co-expression modules closely related to spike germination traits. Two of the modules associated with physiological indicators were analyzed in depth, and nine core genes were finally predicted. Further functional annotation revealed four key transcription factors involved in the regulation of dormancy and germination processes: *gene LOC110698065*, *gene LOC110696037*, *gene LOC110736224*, and *gene LOC110705759*, belonging to the *bHLH*, *NF-YA*, *MYB*, and *FAR1* gene families, respectively.

**Conclusions:**

These results provide clues to identify the core genes involved in quinoa spike germination. This will ultimately provide a theoretical basis for breeding new quinoa varieties with resistance.

**Supplementary Information:**

The online version contains supplementary material available at 10.1186/s12864-024-11151-y.

## Background

Quinoa (*Chenopodium quinoa* Willd.) is a summer annual dicotyledonous plant in the Chenopodiaceae family [1, 2]. It is a highly drought-resistant, salt-tolerant, and cold-resistant C3 crop [3–5]. Quinoa was first domesticated in the Andean region of South America 7,000 years ago [6] and then spread from South America to the rest of the world [7]. According to the International Food and Agriculture Organization (FAO), quinoa is the only plant that meets all the basic nutritional needs of humans and provides nutrients [8] such as antimicrobial, anticancer, antioxidant, and anti-obesity properties. Quinoa seeds are gluten-free [9], making them ideal for celiacs and diabetics [10–12]. Quinoa seeds also contain fiber, vitamins and minerals such as calcium, zinc, magnesium and iron [13]. Due to these nutritional properties, quinoa is also known as a “superfood” [14]. The United Nations declared 2013 as the International Year of Quinoa, further increasing attention and interest in quinoa [15].

Spike germination is a phenomenon in which grains encounter rain before harvest, breaking the dormancy of the seeds and germinating directly on the spike [16, 17]. The most frequent cereals are wheat, barley, rice and sorghum [18]. The occurrence of spike germination can seriously affect food security issues [19]. In China, the United States, Russia, and Canada have begun to mitigate the damage caused by the occurrence of spike germination through breeding methods [20]. Multiple environments and genetics are the main factors that mainly affect spike germination, which increases the dormancy rate of seeds when the mother plant is under colder conditions during the maturity stage of the seeds [21]. It has been shown that kernel colour and seed coat affect dormancy [22],and red-grain wheat varieties tend to have better resistance to spike germination than white-grain wheat, and resistance genes on chromosomes 3A, 3B and 3D were shown to be associated with red seed coat [23]. However, some other studies have shown that resistance genes can also be obtained from white wheat [24]. In addition, degradation of storage starch is one of the energy-providing processes during spike germination [25],and requires the synergy of several enzymes, of which α-amylase is considered to be the main one [26]. Increasing attention is being paid to spike germination, which is a complex phenomenon influenced by genetic, physiological and environmental factors [27].

The phenomenon of spike germination was noticed as early as the 1980s. Many quantitative trait loci (QTLs) associated with spike germination have been identified and extensively studied [28]. In recent years, scientists have identified several genes related to spike germination in wheat and rice, such as *OsPHS1* to *OsPHS9*, through meta-analysis and large-scale mutant screening. These genes are mostly involved in the synthesis and signaling of plant hormones such as abscisic acid (ABA) and gibberellin (GA) [29, 30]. In rice, it has been shown that mutations in the *OsPHS1* to *OsPHS7* genes lead to a spike germination phenotype, where *OsPHS8* affects ABA signaling by regulating the accumulation of small-molecule sugars in the endosperm, whereas *OsPHS9* encodes a CC-type glutaredoxin, which is unique to higher plants, and integrates reactive oxygen species signaling and ABA signaling by binding to *OsGAP*, the ABA receptor interacting protein, thus regulating the spike germination process in rice [31]. Recent studies have also revealed that genes such as *OsFTIP1*, *OsMFT1*, and *OsMFT2* are involved in the spike germination regulatory mechanism in rice, affecting seed dormancy by regulating the expression of genes for key enzymes of ABA and GA biosynthesis [32]. In addition, the *PHS1* to *PHS9* genes in Arabidopsis have been shown to play an important role in seed dormancy and germination. These genes control seed dormancy by regulating ABA and other phytohormone signaling pathways [33].

Weighted gene co-expression network analysis (WGCNA) is an algorithm for mining module information from gene chip expression data, which clusters genes from similar gene expression patterns to form modules and analyses module-specific features [34, 35]. Today, WGCNA is the most commonly used method to identify patterns of correlation between genes [36]. It is becoming more and more widely used in plant research applications: Zhu et al., in order to know the different co-expression modules of rice under salt stress, analysed 457 core DEGs by using WGCNA and successfully obtained three modules, concluding that the resultant three modules were positively correlated with rice salt stress, suggesting that the genes in the modules positively regulated the salt tolerance of rice [37]. Li et al., by analysing the transcriptomes of Cd-treated different maize varieties for WGCNA analysis, divided them into 37 different gene network modules and identified five candidate genes in maize kernels that respond to cadmium stress, which provides a useful dataset for the intrinsic mechanism of cadmium accumulation in maize [38]. WGCNA can efficiently identify collections of genes with similar expression patterns, which in turn reveal the correlation between these collections of genes and the sample phenotype. By mapping the regulatory network among genes, this technology helps to identify key regulatory genes. Meanwhile, WGCNA has been applied in various crop research and has achieved significant results, verifying its effectiveness and practicality.

Spike germination characteristics of quinoa, a crop with important economic value, directly affect yield and quality. Different quinoa lines show significant phenotypic variation during spike germination, which may involve different regulatory mechanisms and hub genes. However, although it is known that some genes may be involved in the regulation of spike germination in quinoa, the specific regulatory mechanisms and hub genes have not been fully elucidated. Therefore, this study aimed to systematically compare the gene expression changes of different quinoa lines during spike germination by transcriptome sequencing and WGCNA to reveal the potential regulatory networks and hub genes. This not only helps to understand the molecular basis of quinoa spike germination but also provides valuable genetic resources for quinoa breeding and trait improvement. Through this study, we expect to fill the existing knowledge gap and provide strong scientific support for the genetic improvement and yield enhancement of quinoa. In this study, two quinoa lines, Dianli-222 (sensitive) and Dianli-654 (resistant), were selected and labeled as group F (sensitive) and group S (resistant), respectively. By integrating data on starch, sucrose, alpha-amylase, and phenolic differential metabolites, a co-expressed gene network was constructed using WGCNA analysis. During this process, we focused on two key modules and identified the core genes of each module: the *MYB* transcription factor family, *NF-YA* transcription factor family, and *bHLH* transcription factor family. These transcription factor families play important roles in dormancy and germination. Therefore, the results of this study have reference value for quinoa breeding under conditions of spike germination.

## Results and analyses

### Transcriptomic analyses

The present study contained 18 samples (two strains were sampled in three time periods, and each time period was repeated three times) with raw reads ranging from 54,021,628 to 66,227,338 and filtered clean reads ranging from 51,851,606 to 64,042,540. The percentage of total bases in the reads with Q-Phred mass scores of not less than 20 and 30 was both above 90%. In addition, for these high-confidence data, the sum of the two bases, guanine (G) and cytosine (C), as a percentage of the total number of all nucleotides ranged from 42.73% to 43.95%, showing good sequence characterization and consistency (Fig. S1). A total of 154.51 Gb of high-quality sequencing data were obtained, and the sequencing reads of each sample were successfully aligned to the genome with an alignment efficiency higher than 88%. These results indicate that the quality of transcriptome sequencing is sufficient for further analysis.

### Gene expression analyses

**Fig.1:**
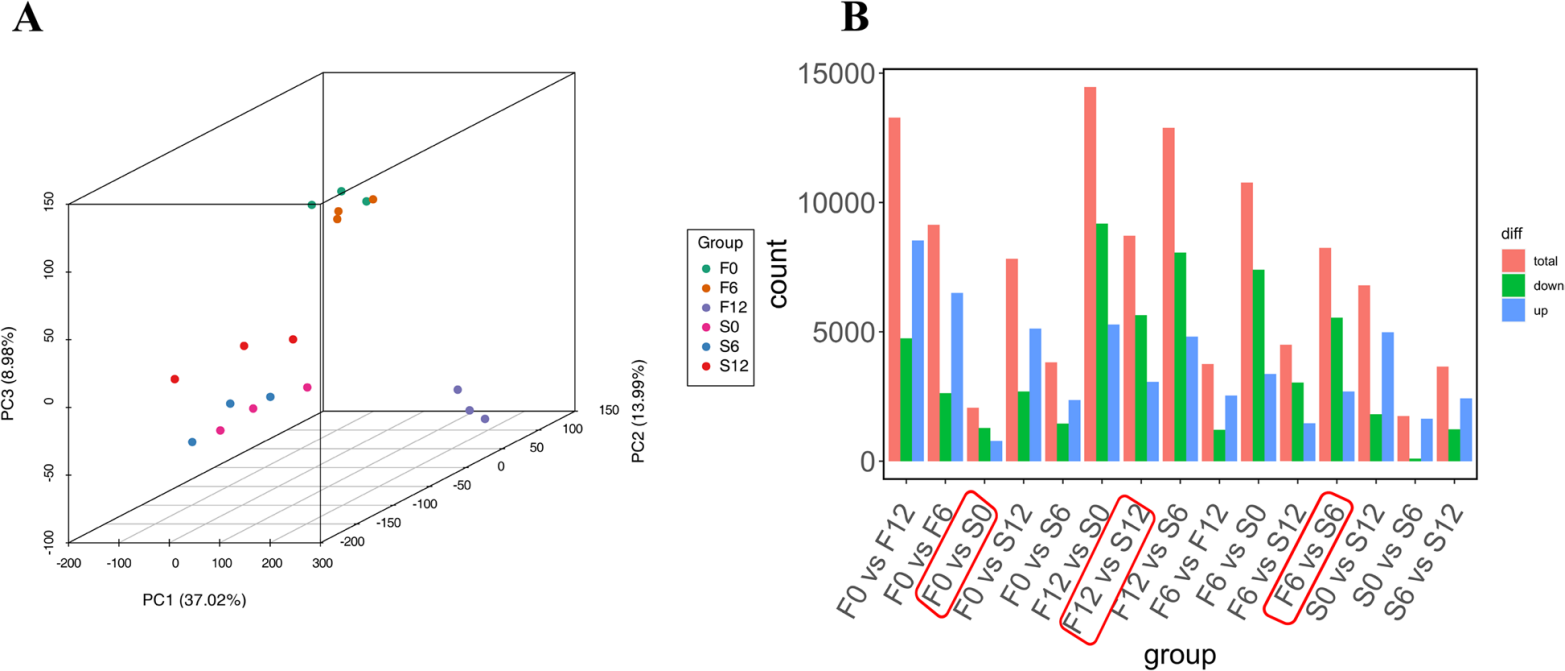
Global view of gene expression profiling. (**A**) Principal component analysis (PCA) of the RNA-seq data. (**B**) Bar graph of differentially expressed genes. Note: (**A**) PC1 is the first principal component, PC2 is the second principal component and PC3 is the third principal component. (**B**) Horizontal coordinates represent groups and vertical coordinates represent the number of genes

PCA analysis based on transcriptome data (Fig. 1A) was performed to know the differences between quinoa samples under high humidity treatment. The first principal component is 37.02%, the second principal component is 13.99%, and the third principal component is 8.98%. The samples are tightly clustered within groups and sparsely dispersed between groups, and the experimental data are stable and reliable for subsequent experimental analysis. It can be seen from the figure that the differentially expressed genes of both strains are continuously up-regulated over time. By visualizing the blue bars, we can clearly observe that the number of up-regulated genes among the differentially expressed genes shows a significant and continuous increase, while the number of down-regulated genes shows a gradual decrease. This phenomenon strongly suggests that up-regulated genes may play a more critical and dominant role, and their dynamics are important for understanding the mechanism of quinoa spike germination (Fig. 1B). F0 vs S0, with 2,066 differential genes, 783 up-regulated and 1,283 down-regulated; F6 vs S6, with 8,250 differential genes, 2,697 up-regulated and 5,553 down-regulated; and F12 vs S12, with 8,711 differential genes, 3,065 up-regulated and 5,646 down-regulated.

### Differential gene GO and KEGG analysis

**Fig.2:**
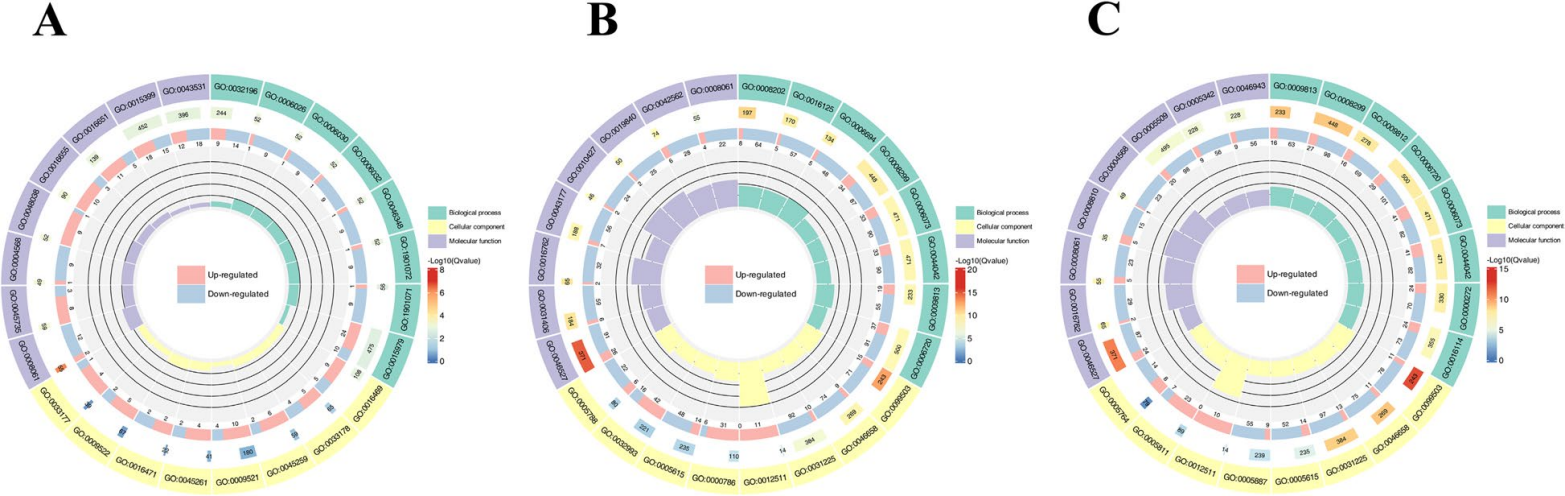
Differential Gene GO Enrichment Circle Map. **A** F0 vs S0. **B** F6 vs S6. **C** F12 vs S12. Note: From the outside to the inside, the first circle shows the entries of the three main GO categories; the second circle show the number of background genes in that category and the q-value; the third circle displays the bar graph of the proportion of up- and down-regulated genes; and the fourth circle presents the Rich Factor value (the number of foreground genes in the category divided by the number of background genes) for each category, with the background auxiliary line indicating 0.2 per cell

**Fig.3:**
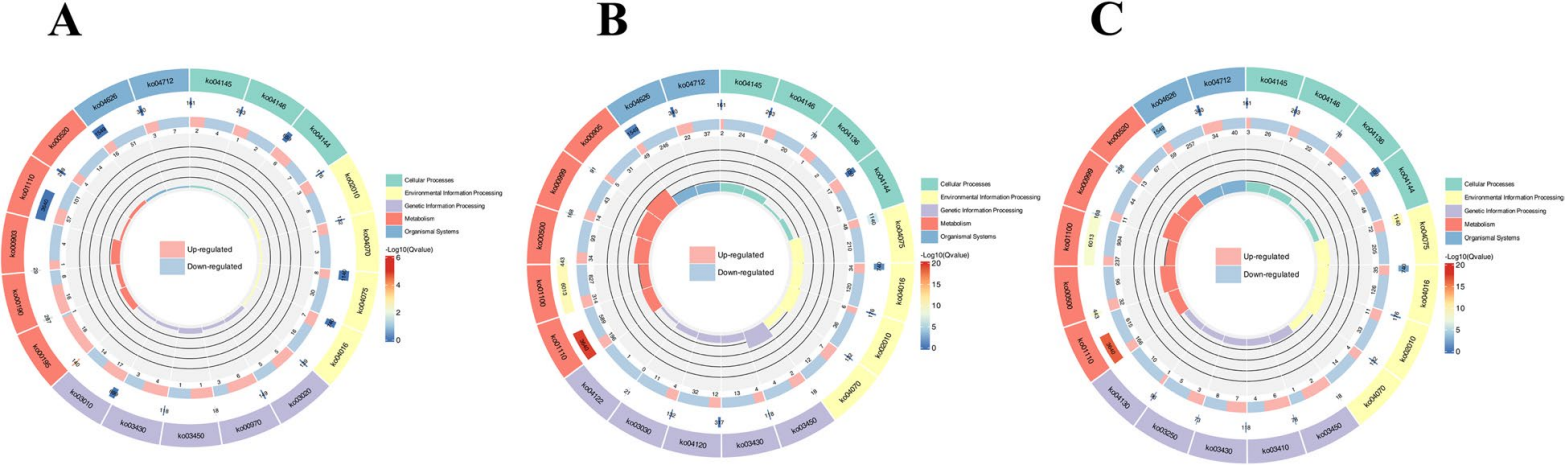
Differential gene KEGG circle map. **A** F0 vs S0. **B** F6 vs S6. **C** F12 vs S12. Note: From the outside to the inside, the first circle is the KEGG_level_1 entry; the second circle is the number of background genes in that classification as well as the q-value; the third circle is the bar graph of the proportion of up- and down-regulated genes; and the fourth circle is the Rich Factor value for each classification (the number of foreground genes in the classification divided by the number of background genes), with each cell of the background auxiliary line indicating 0.2

After screening the differential genes, the distribution of the differential genes in Gene Ontology was investigated by enrichment analysis to elucidate the manifestation of sample differences in gene function in the experiment. GO is classified into Molecular Function, Biological Process, and Cellular Component. Differential genes are mainly involved in chitin binding (GO:0008061), glucosyl transferase activity (GO:0046527), secretory vesicle (GO:0099503), flavonoid biosynthetic process (GO:0009813), and isoprenoid biosynthetic process (GO:0008299) (Fig. 2A-C). We further analyzed differential gene enrichment in the KEGG pathway. The results showed that the biosynthesis of secondary metabolites, amino sugar and nucleotide sugar metabolism, plant hormone signal transduction, brassinosteroid biosynthesis, and starch and sucrose metabolism were significantly enriched (Fig. 3A-C).

### Differences in seed germination between sensitive and resistant quinoa at different time points

**Fig.4:**
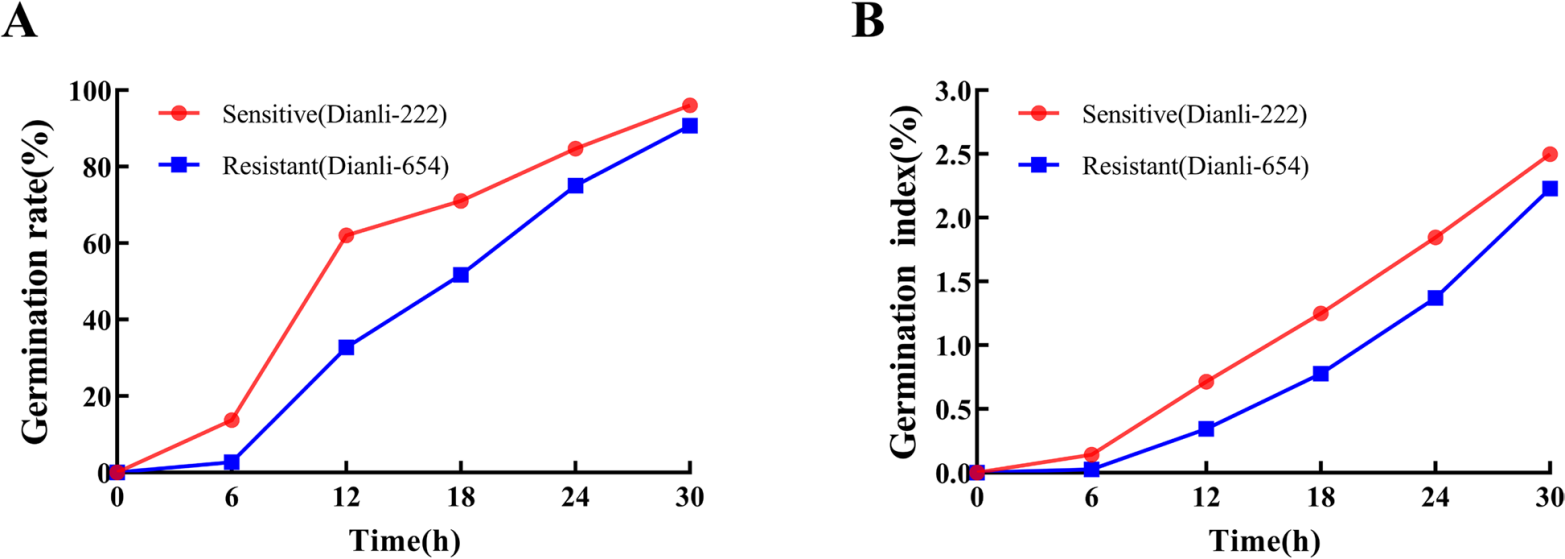
Comparison of germination rate and germination index of different quinoa lines. **A** germination rate. **B** germination index

In this study, in order to deeply explore the differences in spike germination behavior between the two quinoa lines Dianli-222 and Dianli-654, we systematically determined the germination rate and germination index of these two lines at different time periods (Fig. 4A-B). Through the experimental observations, it was clearly observed that the germination rate and germination index of both lines showed a significant increasing trend with time and reached the maximum value at 30 h. It is particularly noteworthy that Dianli-222 and Dianli-654 showed a large difference in the degree of change in germination rate and germination index during the early stages of germination, i.e., from zero to six hours and from six to 12 h. This finding indicates that the differences in physiological responses of the seeds were particularly significant during these two time periods, and therefore we chose these time points as standardized sampling times to more accurately assess and compare the differences in the performance of the two lines under spike germination conditions. By analyzing germination data at these key time points, this study revealed that Dianli-222 and Dianli-654 differed significantly in their biological behaviors and response patterns during early spike germination. These data are important for understanding the genetic and physiological mechanisms of spike germination and can provide a scientific basis for breeding for spike germination resistance traits in quinoa.

### Changes in physiological parameters of quinoa under spike germination

**Fig.5:**
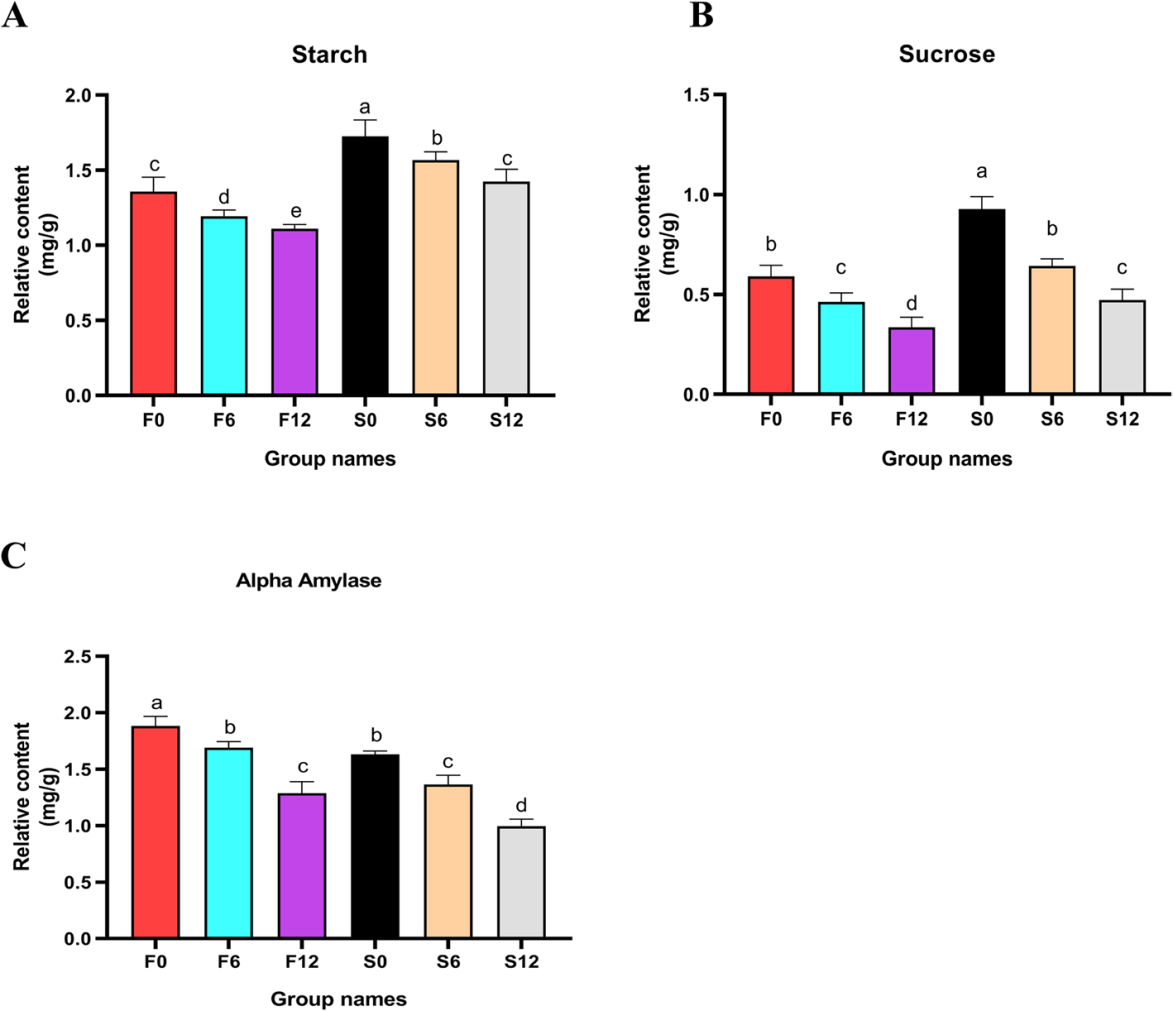
Physiological changes in the germination of quinoa spike. **A** Starch. **B** Sucrose. **C** Alpha-amylase. Note: Identical letters (a-c) indicate no significant differences (*P* > 0.05), and groups with different letters indicate significant differences (*P* < 0.05)

In order to study the physiological response of quinoa spike germination, the physiological indexes of starch, sucrose, and α-amylase were determined. In the process of spike germination, it was noted that the starch and sucrose contents of both sensitive and resistant strains showed a downward trend. However, it was obvious from the data that the initial content of starch and sucrose in the resistant strain was significantly higher than that in the sensitive strain, which implied that the resistant strain had more abundant energy reserves in the early stage of spike germination. In addition, the starch content of the two strains after treatment was lower than that of their respective control groups (Fig. 5A-B). In the study of α-amylase activity, an unexpected phenomenon was found: the strains with higher starch content had less α-amylase content, indicating an inverse relationship between the two (Fig. 5C). This discovery provides a new perspective for understanding the physiological mechanism of quinoa spike germination and may provide a scientific basis for cultivating quinoa varieties with greater resistance to spike germination.

### Construction of quinoa weighted gene co-expression network and module identification

**Fig.6:**
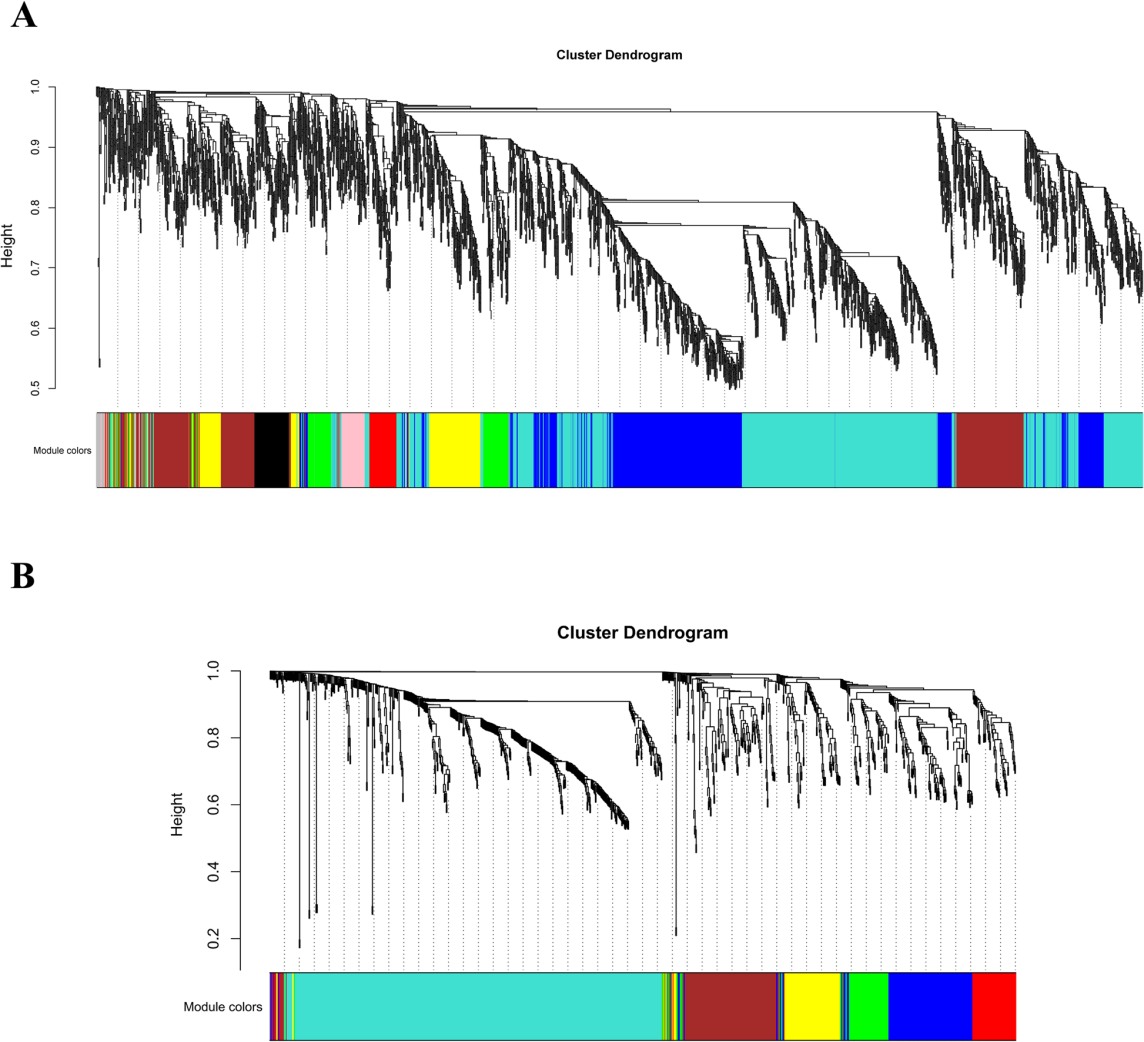
Tree clustering diagram WGCNA. **A** Hierarchical clustering tree diagram of quinoa spike germination mRNA modules. Each individual colored row represents a coding module containing a set of highly connected genes. **B** Hierarchical clustering tree diagram of quinoa spike germination metabolite modules

A total of 9519 genes were obtained through screening and filtering (Table. S1). Cluster analysis was performed on 18 samples. The results showed that there were no outlier samples in the data and all 18 samples were clustered (Fig. S2). The selection of soft threshold (Power) is a key step in network construction. When the soft threshold is 14, the scale-free topological fit index R^2^ > 0.8 and the average connectivity gradually converges to 0 (Fig. S3). The scale-free network distribution is fully conformed (Fig. S4). The network was established according to the determined soft threshold (β = 14), and the clustering tree was constructed relying on the expression correlation between genes (Fig. 6A), and nine modules were finally constructed. Different colours represent different modules, with the turquoise-coloured module having the highest number of genes (3431) and the pink-coloured module having the lowest number of genes (249) (Fig. S5). On the other side, 1538 metabolites were obtained (Table. S2), and the data were stable and reliable as shown from the clustering diagram (Fig. S6). When a soft threshold (β = 20) was constructed for the network (Fig. S7), the final 7 modules (Fig. 6B), among which the turquoise colour module had the highest number of genes (540) (Table. S3).

### Identification of specific modules related to quinoa spike germination

The correlation of each characterized gene with sample treatment conditions was calculated, and two gene co-expression modules were identified (at |r|> 0.50, *p* < 0.05) (Fig. S8A). The Red module (r = 0.97, *p* = 7.3e-12) was positively correlated with the sensitive material, and the Blue module (r = −0.97, *p* = 7.3e-12) was negatively correlated with resistant material. The core genes of the Red module, such as *gene LOC110718721*, *gene LOC110718730*, and *gene LOC110718724*, were identified (Fig. S9). In order to know the relationship between genes and physiological traits, we calculated the correlation between modular eigenvalues (ME) and physiological traits (Table. S4). A total of two co-expression significant modules were identified that were specifically associated with quinoa spike germination. The Blue module (r = 0.80, *p* = 0.00006) showed a positive correlation with Alpha-amylase, and the Green module (r = −0.74, *p* = 0.00042) showed a negative correlation with Sucrose (Fig. S8B). Overall, there were significant differences between genes and physiological indicators in the 2 modules (Fig. S10). By our observation of the degree of variation in the differential metabolites across subgroups, significant differences were noted for phenolics, which are hypothesized to play an important role in the onset of spike germination. By calculating the correlation (Fig. S8C) between genes and phenolic metabolites (Table. S5), we found that the positive and negative correlations were mainly concentrated in the Red module (|r|> 0.90, *p* < 0.01), and we found that some of the core genes belonged to the *C3H*, *bZIP*, *Trihelix*, and *MYB*-related gene families. By calculating the correlation between phenolic metabolites and physiological traits (Fig. S8D), it was found that the Brown module (r = 0.78, *p* = 0.00011) was positively correlated with presenting sucrose. These above modules safeguarded the subsequent research and mining of core genes.

### Enrichment analysis of genes in modules of interest

**Fig.7:**
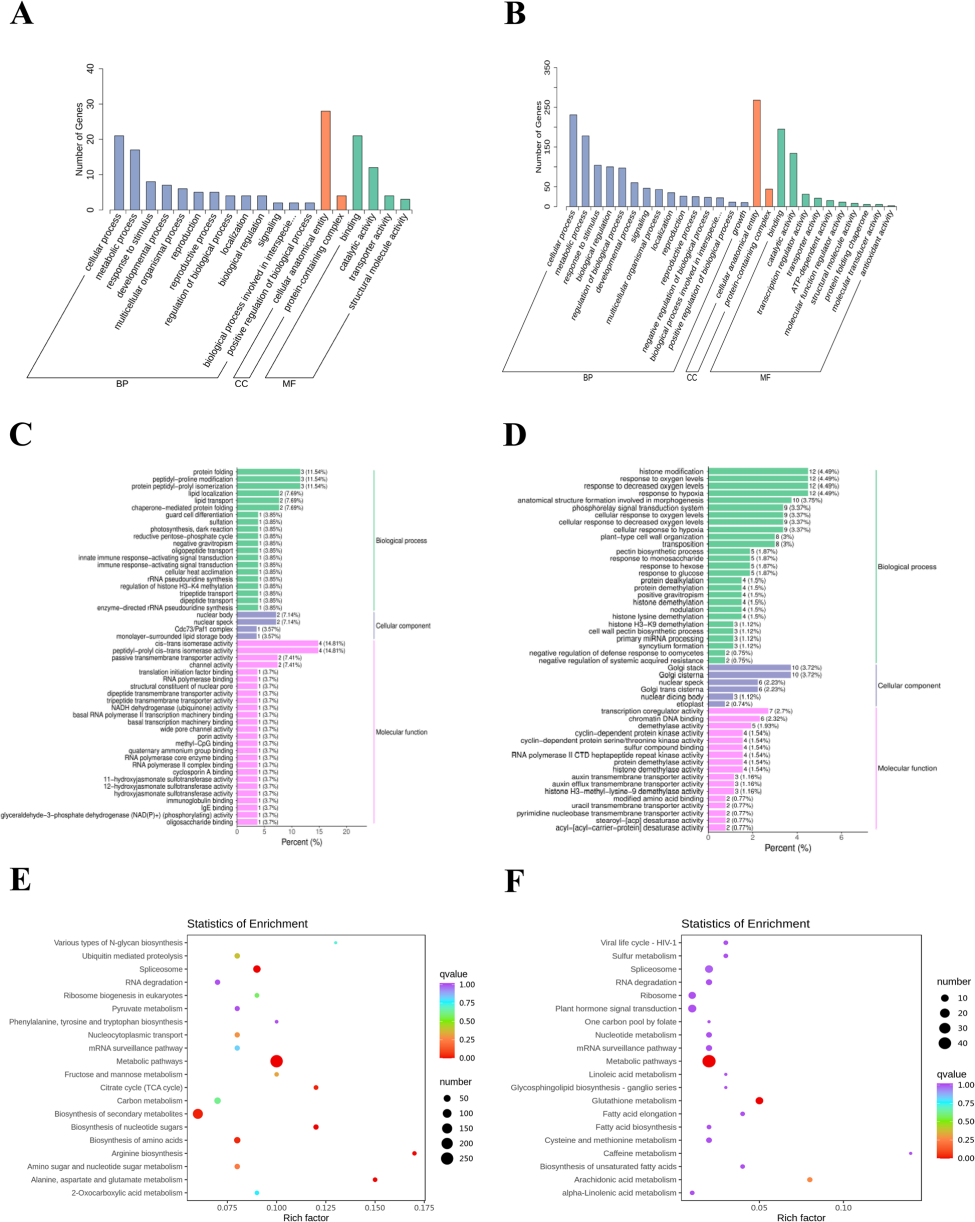
Blue (**A**, **C**, **E**) and Green (**B**, **D**, **F**). GO annotation (**A**, **B**), GO enrichment (**C**, **D**) and KEGG enrichment (**E**, **F**) analyses

To explore the functional classification and metabolic pathways of the responsive genes in different quinoa lines, we performed GO analysis of the genes in the red module and found that they were mainly enriched in pyridine-containing compound metabolic process (GO:0072524), tRNA aminoacylation for protein translation (GO:0006418), amino acid activation (GO:0043038), and tRNA metabolic process (GO:0006399) (Fig. S11). In order to have a more intuitive understanding of the main functions of the modular genes, two specific modular genes highly correlated at different physiological levels were annotated into the KEGG database and were found to be mainly enriched in Aminoacyl-tRNA biosynthesis and Biosynthesis of secondary metabolites (Fig. S12). To further understand the functional classification and metabolic pathways of different physiological indicator genes for quinoa spike germination, we analyzed the GO enrichment of genes in the blue and green modules. The genes in the blue module were classified into 163 significantly enriched GO terms, including 90 Biological processes, 32 Cellular components, and 41 Molecular functions (Fig. 7A, Table. S6), which were mainly enriched in peptidyl-prolyl cis–trans isomerase activity (GO:0003755), cis–trans isomerase activity (GO:0016859), protein peptidyl-prolyl isomerization (GO:0000413), peptidyl-proline modification (GO:0018208), and protein folding (GO:0006457) (Fig. 7C, Table. S7). The genes in the green module were classified into 1764 significantly enriched GO terms, including 1021 Biological processes, 312 Cellular components, and 431 Molecular functions (Fig. 7B, Table. S8), which were mainly enriched in response to hypoxia (GO:0001666), response to decreased oxygen levels (GO:0036293), response to oxygen levels (GO:0070482), chromatin DNA binding (GO:0031490), and Golgi cisterna (GO:0031985) (Fig. 7D, Table. S9). The blue module focuses on Amino sugar and nucleotide sugar metabolism (Ko00520), Alanine, aspartate and glutamate metabolism (Ko00250), Biosynthesis of nucleotide sugars (Ko01250), and Citrate cycle (TCA cycle) (Ko00020) were enriched (Fig. 7E, Table. S10). The green module was mainly enriched in Arachidonic acid metabolism (Ko00590), Glutathione metabolism (Ko00480), and Metabolic pathways (Ko01100) (Fig. 7F, Table. S11).

### Identification of core genes in the significant co-expression module of quinoa spike germination and construction of gene interaction network

**Fig.8:**
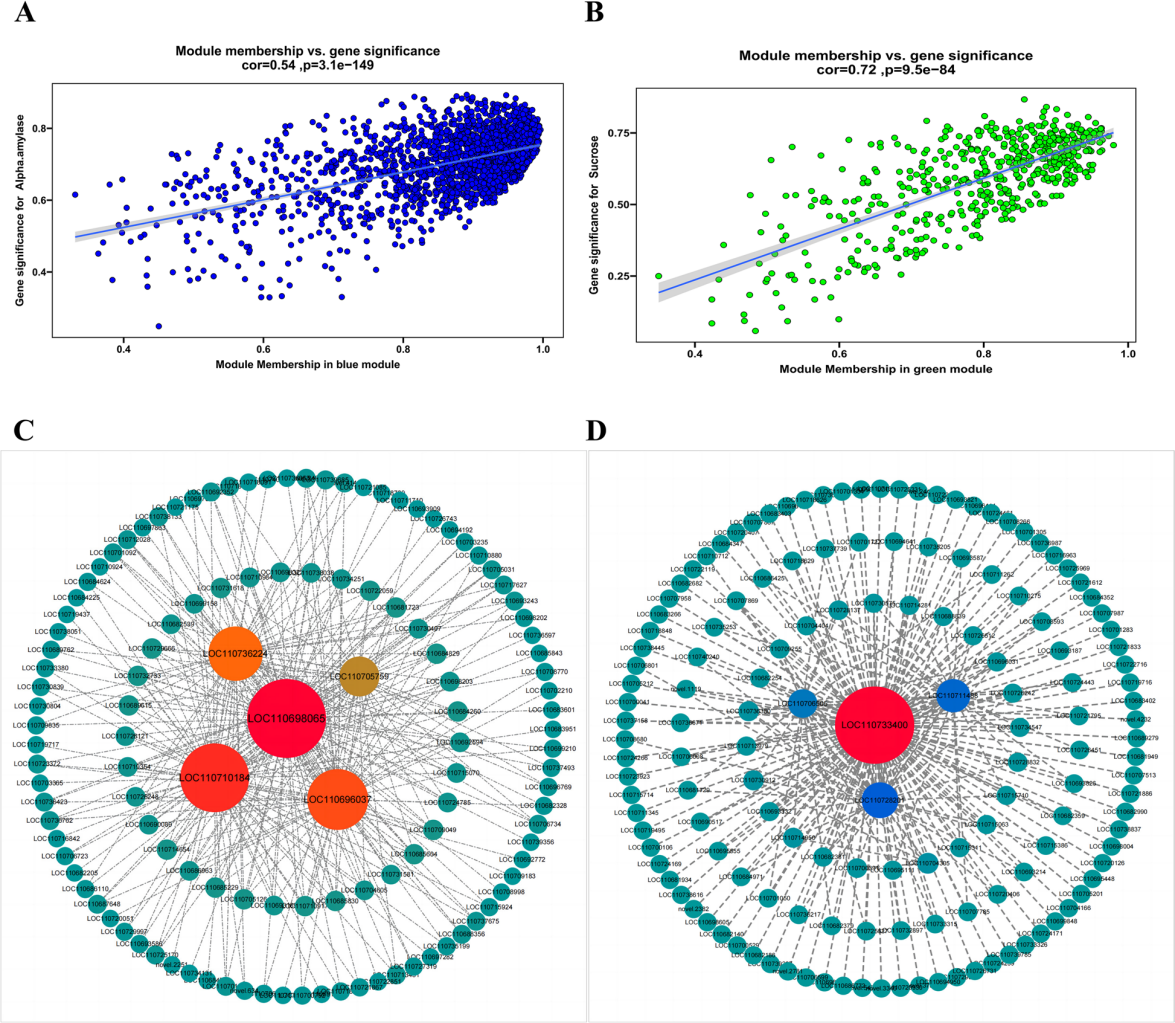
Scatter plots of gene significance (GS) versus module membership (MM) in Blue (**A**) and Green (**B**) modules. Candidate hub genes for Blue (**C**) and Green (**D**) obtained from interaction network analysis with known core genes

Scatter plots of GS and MM values were plotted for the blue and green modules (Fig. 8A-B). Key genes were screened by setting |GS| to > 0.7 and |MM| to > 0.8. We obtained 985 and 111 pivotal genes in the blue and green modules, respectively (Fig. 8C-D). The Analyze Network in Cytoscape 3.10.0 was subsequently used to select the top degree-ranked genes as hub genes. Finally the blue module identified *genes LOC110698065*, *LOC110710184*, *LOC110696037*, *LOC110736224*, *and LOC110705759*. The green module identified *genes LOC110733400*, *LOC110728201*, *LOC110711488*, *and LOC110706505*.

Protein sequence comparison of the module hub genes through the plantTFDB website revealed that the core genes in the blue module belonged to the *MYB* transcription factor family, *bHLH* transcription factor family, *NF-YA* transcription factor family, and *FAR1* transcription factor family, respectively. Most of the core genes in the blue module belonged to the *MYB* transcription factor family, *LOB* transcription factor family, *WRKY* transcription factor family, *AP2/ERF-ERF* transcription factor family, and the functions of the key candidate core genes were further understood by annotating them on the TAIR website (Table. S12).

### Real-time fluorescence quantitative PCR validation

**Fig.9:**
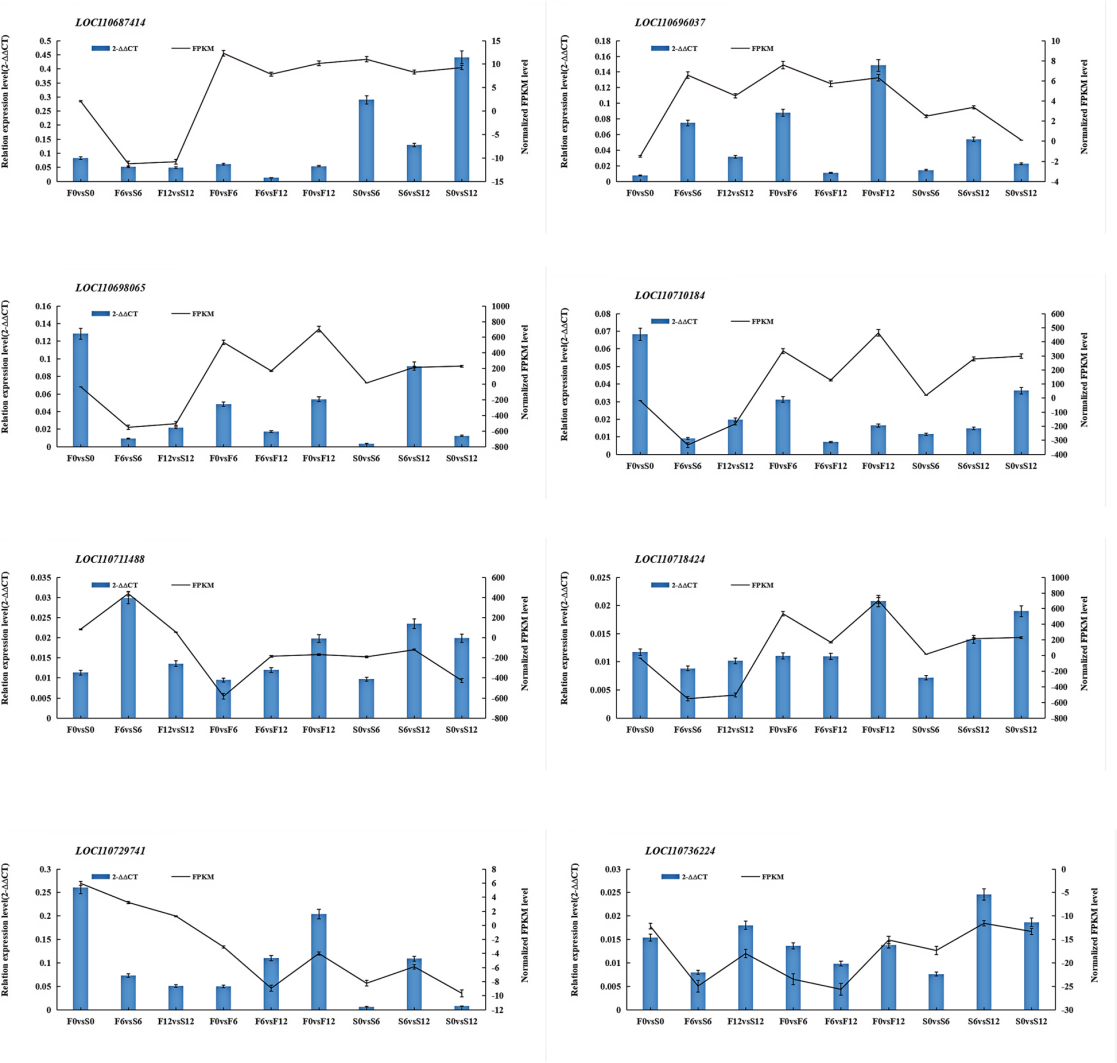
Validation of the transcription levels for selected DEGs via RT-qPCR

In order to determine the authenticity and reliability of the differential expression levels of the transcriptome data, randomly selected genes from the core genes in the key modules and the transcriptome were analyzed by qRT-PCR (Fig. 9) (Table. S13). The results showed that all qRT-PCR results were consistent with the expression patterns of RNA-seq data.

## Discussion

Quinoa is rich in nutritional value and loved by the public [39],but sprouting of spikes reduces its nutritional value, yield and quality [40]. Spike germination has often been studied through traits such as dormancy [41], seed color [42], germination rate [43], and α-amylase [44]. It has been shown that spike germination tolerance is associated with red kernels [45], and Tamyb10 affects spike germination resistance by activating flavonoid biosynthesis-related genes that regulate kernels to be red [46]. The materials in this study were all red quinoa, and by mining the core genes of the module, we can provide more theories for the study of the molecular mechanism of spike germination. Liu [47] et al. found that growth hormone and ABA are interdependent in dormancy, and interruption of growth hormone signaling releases seeds from dormancy, while vice versa increases dormancy. It was also demonstrated that seed dormancy is related to the uptake of endogenous ABA from seeds [48–50], while ABA synthesis is critically regulated by 9-cis-epoxycarotenoid dioxygenase [51]. Zhang [52] et al. overexpressed wheat α-amylase TaAMY1 and found that elevation of TaAMY1 reduces seed dormancy and enhances ABA resistance, and is associated with α-glucose oligosaccharides and sucrose. Cong [53] et al. increased phosphorus utilization efficiency of crops by planting high phosphorus genotypes while reducing pressure on the environment. These studies have laid the foundation for subsequent selection of resistant varieties, and knowing the core genes of a crop provides a new tool for variety selection.

With the development of sequencing technology, RNA-seq has been widely used in plant analysis [54]. In this study, GO [55] and KEGG [56] were used to analyze the differential genes in the module, which were mainly enriched in amino acids, nucleotides, energy metabolism and glucose metabolism. After that by screening the module core genes with gene function annotation and transcription factor prediction, this experiment learned that most of the core genes are more important for the bHLH transcription factor family, NF-YA transcription factor family, MYB transcription factor family, FAR transcription factor family, WRKY transcription factor family etc.

It is well known that transcription factors are regulatory proteins that are involved in the regulation of crop growth, development, and environmental responses [57]. In our study, *gene-LOC110710184* and *gene-LOC110733400* belonged to the *MYB* family, and Yang [58] et al. identified two Arabidopsis thaliana *MYB* transcription factors, *RVE1* and *RVE2*, and found that they were not only involved in seed germination but also regulated seed dormancy. Sabir [59] et al. investigated 69 sweet cherry genomes from the *MYB* genes and found that *MYB* genes may play an important role in bud dormancy through transcriptomic data. These suggest that the core genes we identified likewise play an important role in quinoa spike germination. *Gene-LOC110696037* and gene-*LOC110736224* belong to the *NF-YA* family. Ding [60] et al. identified and characterized the dormancy specific target gene *paghap2-6* of *mir169*. *Paghap2-6* was identified as a homolog of the *NF-YA* transcription factor, and overexpression in Arabidopsis can increase resistance to exogenous ABA. Recent studies have shown that [61] *NF-YA* subunits play an important role in ABA-mediated responses in plants, and *NF-YA* mutants and overexpressing strains also show ABA-related characteristics in seed germination. Next, we will study the composition, function and evolution of the NF-YA gene family in quinoa. *Gene-LOC110698065* belongs to the *bHLH* family, and the change of *bHLH* family activity may affect the transformation process of seeds from dormant state to germination state. Gao [62] et al. found that tomato SIAN11 regulates flavonoid biosynthesis and seed dormancy by interacting with *bHLH* protein, rather than interacting with *MYB* protein. Liu [63] et al. showed that ODR1 negatively regulated seed dormancy by interacting with the transcription factor *bHLH57* and preventing it from inducing the expression of NCED6 and NCED9 and ABA biosynthesis. These studies also showed that the core genes we identified also played an important role in the germination and dormancy of quinoa spike.

This study focuses on two gene modules that are closely related to physiological processes. In the blue positive correlation module, up-regulation of the expression of the core genes *LOC110698065*, *LOC110710184*, *LOC110696037*, *LOC110736224*, and *LOC110705759* prompted a synchronized increase in the expression of other related genes, suggesting that these genes may synergistically promote the germination process of quinoa spike. In contrast, the high expression of the core genes *LOC110733400*, *LOC110728201*, *LOC110711488*, and *LOC110706505* in the green negative correlation module was accompanied by a decrease in the expression of the other member genes, suggesting that they may play an inhibitory role in the quinoa spike germination process. This finding lays the foundation for an in-depth exploration of the specific functions of these genes and paves the way for our subsequent experiments, in which we will further validate the practical significance of the core genes in quinoa spike germination by means of knockdown [64] and overexpression [65] in order to reveal their precise biological contributions. Research on spike germination is now making remarkable progress in grain crops such as rice and wheat. Liu et al. [66] revealed gene expression changes during rice spike germination by transcriptome analysis. A total of 9,602 differentially expressed genes (DEGs) were identified, and KEGG pathway enrichment analysis revealed that these genes were mainly involved in key pathways such as phytohormone signaling, carbon metabolism, starch and sucrose metabolism, and phenylpropanoid biosynthesis. Enrichment analysis of closely related gene modules for spike germination in quinoa in our study showed major enrichment in key pathways such as aminosugar and nucleotide sugar metabolism, starch and sucrose metabolism, and the citric acid cycle. These results further confirm the general importance of these metabolic pathways in the spike germination of different crops. Notably, our experimental approach is broadly similar to that of Wei et al. [67], who predicted wheat yield-related candidate genes and molecular networks based on a combination of QTL localization and WGCNA, and our results present unique findings. This could be attributed to differences in the selection of our samples, experimental conditions, or data analysis. This further validates the reliability and effectiveness of the experimental method.

Metabolites are not only the direct product of gene expression, but also the relationship between genotype and phenotype. We analyzed the correlation between genes and phenolic acid metabolites. Benincasa [68] et al. have studied that the content of phenolic substances in grain germination will increase significantly. In quinoa, sprouting increases the levels of phenolic compounds, unsaturated fatty acids, γ-aminobutyrate, and carotenoids [69]. In our study, we found that wayn001856, pmn001468, cmzn004594, mwsSmce675, mws0906, xmyn004302, lman002731, and hmtn001120 were highly correlated with quinoa spike germination genes. The differential genes found by analyzing the related specific red modules were mainly enriched for purine metabolism, aminoacyl-tRNA biosynthesis, biosynthesis of amino acids, and purine metabolism. By transcription factor family comparison, the above genes are involved in the occurrence of quinoa spike germination and play a role. We functionally annotated the core genes and found that quinoa spike germination mainly regulates seed dormancy and germination by regulating ABA signaling. The functional annotation of these genes further identified that these core genes play an important role in regulating quinoa spike germination.

## Conclusion

In summary, quinoa responds to spike germination by regulating osmoregulatory substances such as starch, sucrose and α-amylase. In this study, we constructed a WGCNA co-expression network based on transcriptomic data and focused on screening key modules and core genes for physiological indicators and phenolic metabolites. Then, GO and KEGG analyses were carried out. The core genes mainly belonged to the gene families of *bHLH*, *MYB*, *NF-YA*, *FAR1*, and *AP2/ERF-ERF*. Through functional annotation, some of the core genes were found to be closely related to the reported dormancy and germination of seeds. This study lays a solid foundation for an in-depth exploration of the molecular mechanism of spike germination in quinoa and other similar plants and is expected to provide important guidance for breeding quinoa varieties with spike germination resistance.

## Materials and methods

### Plant materials and growing conditions

The sensitive spike germination line (Dianli-222) and the resistant spike germination line (Dianli-654) were provided by the Yunnan Agricultural University Modern Education and Research Base, located at E 102°41′, N 25°20′. The quinoa seedlings were grown in a greenhouse maintained at 24 °C with a 12-h light cycle. The experiment was conducted immediately after 120 days of maturity. Given the difficulty in observing quinoa spikes with numerous seeds covered by sepals, we randomly selected spikelets from the main quinoa spike and brought them back to the laboratory as experimental materials. To simulate a high humidity environment [66], we sprayed distilled water on the spikes of the two different lines we retrieved and continued this operation for two days. When determining the spike germination rate, the number of germinated seeds was observed and counted every six hours. The results showed that the sensitive strain began to germinate partially at six hours, while the resistant strain showed no germination phenomenon. The difference between the two strains was most pronounced at 12 h. At this time, it was considered to be the optimal sampling time, and samples were taken from both strains at zero, six, and 12 h. A total of 18 samples, each with three biological replicates, were frozen in liquid nitrogen and stored in a refrigerator at −80℃ (Wuhan MetWare Biotechnology Co. Ltd., Wuhan, China; https://www.metware.cn). In this study, quinoa lines Dianli-222 (sensitive) and Dianli-654 (resistant) are labeled as group F (sensitive) and group S (resistant), respectively. F0/S0 represents the zero-hour untreated control group, while F6/S6 and F12/S12 represent the six-hour and 12-h treated groups.

## RNA isolation, library construction, and sequencing

In this experiment, six libraries were constructed to represent the seed samples of the two strains and their three repetitive sequences. Eighteen samples were sent to Wuhan Metware Biotechnology Co., Ltd. for transcriptome and metabolome profiling. The transcriptome analysis process includes RNA extraction, RNA detection, and RNA library construction. In this study, samples taken from two quinoa lines at three spike germination stages were used for analysis. The CTAB method used in this project were used to extract RNA [70]. The kit used was the Hieff NGS® Ultima Dual-mode mRNA Library Prep Kit. It was first determined by agarose gel electrophoresis; then, prior to library construction, the concentration and integrity of RNA were examined using a Qubit 2.0 fluorometer (Thermo Fisher Scientific, Waltham, MA, USA) and an Agilent 2100 Bioanalyzer (Agilent Technologies, Santa Clara, CA, USA) to detect RNA concentration and integrity. The library construction process includes RNA fragmentation, first- and second-strand cDNA synthesis, end repair, adenylation and aptamer ligation. Afterwards, 250–300 bp cDNA fragments were selected using the AMPure XP system and post-processed for size selection using the USER enzyme. Finally, after amplification and purification of the product by PCR, the library quality assessment was completed and sequenced on March 24, 2023; after library construction was completed, the library quality was initially quantified using the Qubit dye method. The insert size of the libraries was examined using a fragment analyzer, and the insert size met the expectation before proceeding to the next step of the experiments. The effective concentration of the libraries was accurately quantified by the Q-PCR method (effective concentration of the libraries > two nM), and the library check was completed. When the libraries were constructed to meet quality control standards, they were sequenced on the Illumina platform. The sequencing was performed using paired-end technology with a read length of 150 base pairs (bp) [71]. Fastp v0.19.3 [72] was used to process the raw reads based on the sequencing quality. Reads with adapter sequences, nitrogen content exceeding 10% of the alkali bases of the read, or bases of low quality (Q ≤ 20) exceeding 50% of the read were removed; both paired reads were removed in the latter two cases to obtain clean data. The reference genome used in this project was GCF_001683475.1_ASM168347v1_genomic.fna (weblink address: https://www.ncbi.nlm.nih.gov/genome/?term=quinoa). HISAT v2.1.0 [73] was used to build an index, compare clean reads with the specified reference genome, and obtain mapped data. Mapped data were obtained for subsequent structural level analysis and expression level analysis. StringTie v1.3.4 [74] was used to predict new genes, and FeatureCounts v1.6.2 [75] was used to calculate the gene alignment. Differential expression analysis between groups was performed using DESeq2 v1.22.1 [76], and *p*-values were corrected using the Benjamini–Hochberg method. After the analysis of variance, multiple hypothesis testing correction for hypothesis testing probability (*P* value) was also required to obtain the False Discovery Rate (FDR) using the Benjamini–Hochberg method. Differential genes were screened for |log2Fold Change|≥ 1 and FDR < 0.05.

## Metabolite extraction and qualitative and quantitative analysis

In this study, the biological samples were placed in a lyophilizer (Scientz-100F) using vacuum freeze-drying technique, and then the samples (30 Hz, 1.5 min) were ground into powder using a grinder (MM 400, Retsch). Then, 50 mg quinoa seed sample powder was weighed using an electronic balance (MS105D Μ), and 1200 μL −20 °C pre-cooled 70% methanol aqueous internal standardized extract was added (less than 50 mg was added at the rate of 1200 μL extractant per 50 mg of sample). Vortexing was performed every 30 min for 30 s for a total of six times. After centrifugation (12,000 rpm, three min), the supernatant was aspirated, and the sample was filtered through a microporous membrane with a pore size of 0.22 μm and stored in an injection vial for UPLC-MS/MS analysis. Using ultra-performance liquid chromatography tandem mass spectrometry (UPLC-MS/MS) technology, combined with the self-built database mwdb (software database), we successfully integrated the data of a variety of target metabonomics and quantitatively identified 1538 metabolites [77]. We screened out differential metabolites based on VIP ≥ 1, equivalent change ≥ 2, and equivalent change ≤ 0.5, which provided valuable data resources for subsequent research [78].

## Germination measurement

The germination rate and germination index were recorded for Dianli-222 and Dianli-654 according to Eqs. (1 and 2) [79] as follows:

1 $$\mathrm{Germination}\;\mathrm{rate}\;\left(\%\right)=\frac{\mathrm{Number}\;\mathrm{of}\;\mathrm{germinated}\;\mathrm{seeds}}{\mathrm{Total}\;\mathrm{number}\;\mathrm{of}\;\mathrm{tested}\;\mathrm{seeds}}\times100\%$$

2 $$\mathrm{Germination\;index}\left(\%\right)=\sum\frac{\mathrm{Gt}}{\mathrm{Dt}}\times100\%$$

Gt is the number of germinations at time t, and Dt is the corresponding days to germination.

## Determination of starch, sucrose and α-amylase content

Physiological indices related to starch content, sucrose content and α-amylase content during the germination of quinoa ears were determined using the Starch Content Determination Kit, Sucrose Content Determination Kit and α-Amylase Content Determination Kit (Wuhan Puyinte Bioengineering Co., Ltd., http://www.pytbio.com). The experiments and calculations were performed strictly according to the manufacturer's instructions.

## Construction of WGCNA co-expression network

Co-expression network analysis was performed using the WGCNA package in R version 4.1.1 [80]. Genes with low expression and no change were filtered, and the top 80% of expression variants in the samples were screened using the Genefilter package in R. A total of 9,519 genes were finally identified. Then, pickSoftThreshold in the WGCNA package was used to calculate the weight values so that the network conformed to the scale-free network distribution. The optimal soft threshold is chosen to be 14. Genes with similar expression patterns were classified into the same module, which was divided into nine modules, and the correlation of each module with physiological traits and related metabolites was calculated.

## Functional annotation and enrichment analysis of modular genes

Correlation coefficients r and *P*-values were calculated for ME values of each module with different traits. r-values responded to the interrelationships of the events and *P*-values responded to the probability of occurrence of the events. In this study, gene ontology (http://www.geneontology.org/) [55] and KEGG [56] analysis (http://www.kegg.jp/kegg/pathway.html) were performed on the modules in order to understand the functioning of the particular module.

## Screening of hub genes

The connectivity of genes within a module represents their regulatory relationships with other genes. Connectivity indicates a gene's role in the module; higher connectivity suggests a stronger regulatory role and potential to become a core gene. Thus, by calculating the KME (module eigengene-based connectivity) values within the module, the top 20 genes were initially selected as candidate core genes. Subsequently, Cytoscape 3.10.0 (https://www.cytoscape.org) was used to screen core genes in the relevant modules. Genes with high connectivity and expression were selected as central genes within the module, and these top-ranked genes were designated as hub genes [81].

## Transcription factors

Transcription factors (TFs) play an important role in regulating various abiotic stress responses. We submitted the protein sequences of the core genes to the plantTFDB database for analysis to obtain the transcription factor families of each module, and then submitted them to the TAIR Arabidopsis website to understand the functions of the core genes.

## Real-time fluorescence quantitative PCR analysis

To verify the accuracy of the module core gene expression and transcriptome, we designed primers for the relevant genes using Beacon Designer 7.9. The eLF-3 gene was selected as the internal reference gene [82]. RT-qPCR was performed using the StepOnePlus instrument (Applied Biosystems, Foster City, CA, USA) with PerfectStart SYBR qPCR Supermix (TransGen Biotech, Beijing, China). The reaction volume was 20 µL (Table.S14), and the thermal cycling conditions were set to 94℃ (30 s), 94℃ (5 s), 60℃ (30 s)for 40 cycles. Finally, the 2 − ∆∆Ct method was used to calculate the relative gene expression levels [83].

## Supplementary Information

Supplementary Material 1.

Supplementary Material 2.

Supplementary Material 3.

Supplementary Material 4.

Supplementary Material 5.

Supplementary Material 6.

Supplementary Material 7.

Supplementary Material 8.

Supplementary Material 9.

Supplementary Material 10.

Supplementary Material 11.

Supplementary Material 12.

Supplementary Material 13.

## Data Availability

The original contributions presented in the study are publicly available. This data can be found in the National Center for Biotechnology Information (NCBI) SRA database under accession number SRP474959. The names of the repository and accession number(s) can be found below: https://www.ncbi.nlm.nih.gov/sra/SRP474959.

## Abbreviations

TF: Transcription factor
WGCNA: Weighted Gene Co-expression Network Analysis;
GO: Gene Ontology
KEGG: Kyoto Encyclopedia of Genes and Genomes
