# Supplementary figures and images for: Comparative transcriptomes and WGCNA reveal hub genes for spike germination in different quinoa lines
Source: BMC Genomics. 2024 Dec 20;25:1231. doi: 10.1186/s12864-024-11151-y (PMC11662621; doi:10.1186/s12864-024-11151-y)

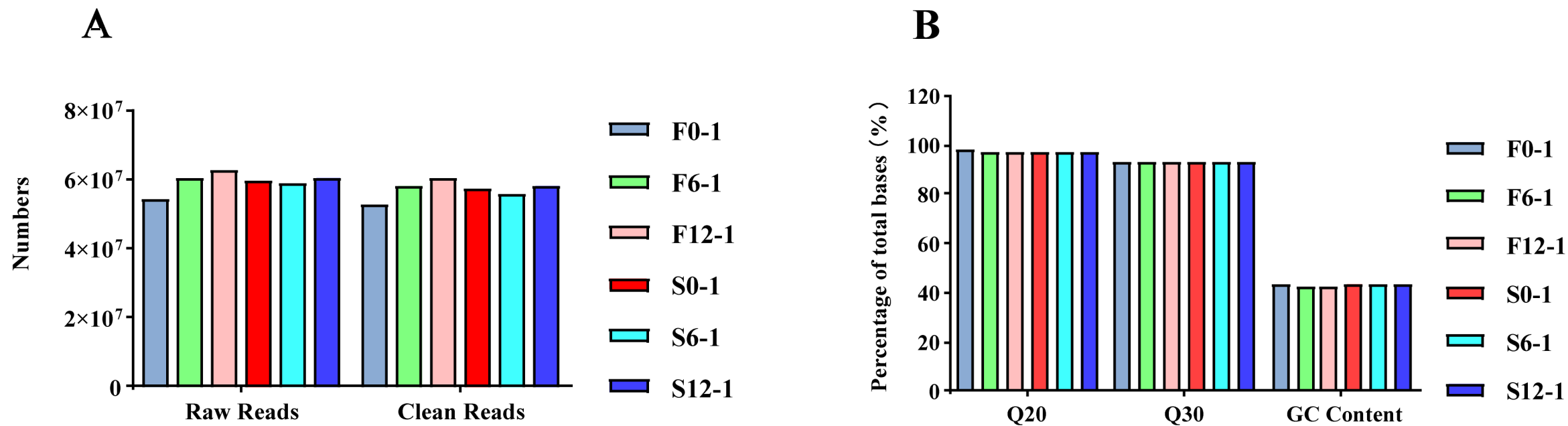

**Fig.S1** Statistical map of sequencing outputs.

(A)Raw Reads and Clean Reads. (B)Q20 and Q30 and GC Content.

Supplement: Supplementary file 1 — Supplementary Material 1. [file 12864_2024_11151_MOESM1_ESM.pdf]

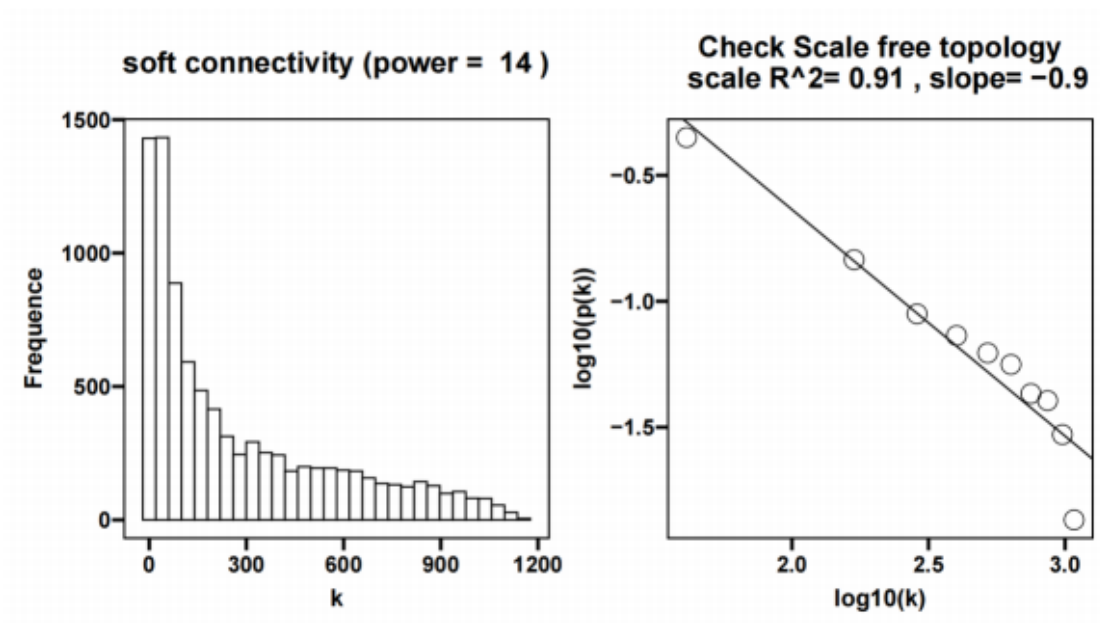

Fig.S4 Scale-free network distributions.

Supplement: Supplementary file 4 — Supplementary Material 4. [file 12864_2024_11151_MOESM4_ESM.pdf]

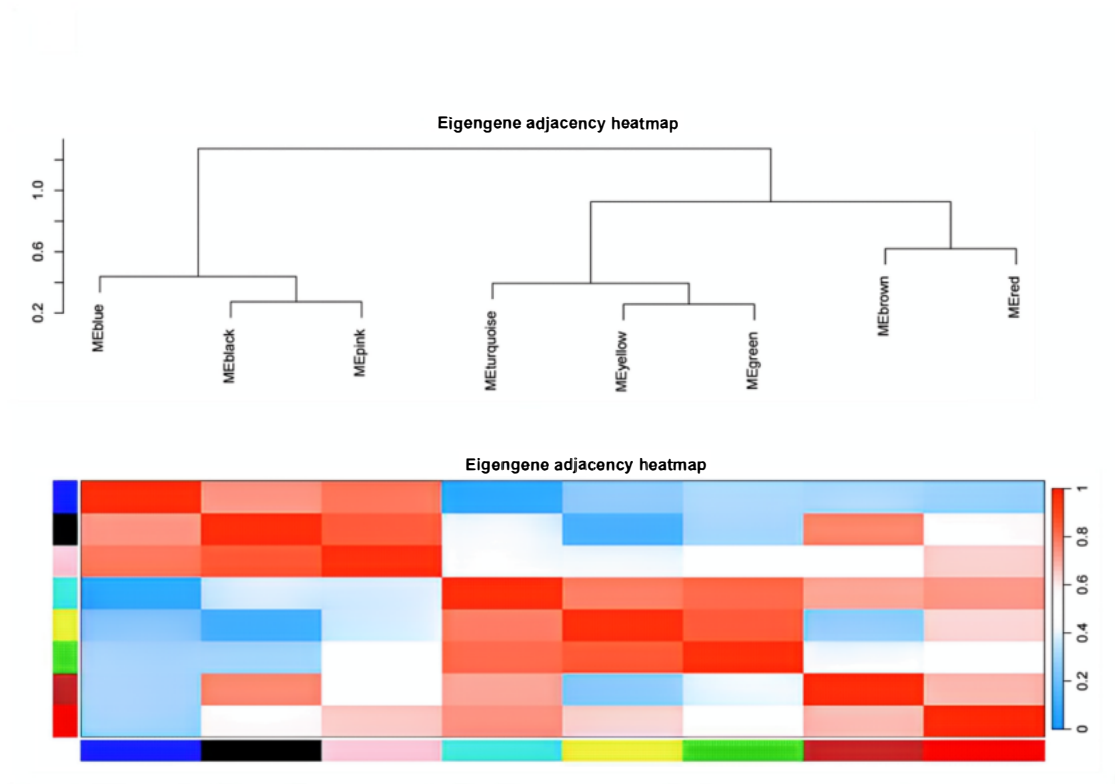

**Fig. S5** Visualisation of feature gene networks representing relationships between modules.

Supplement: Supplementary file 5 — Supplementary Material 5. [file 12864_2024_11151_MOESM5_ESM.pdf]

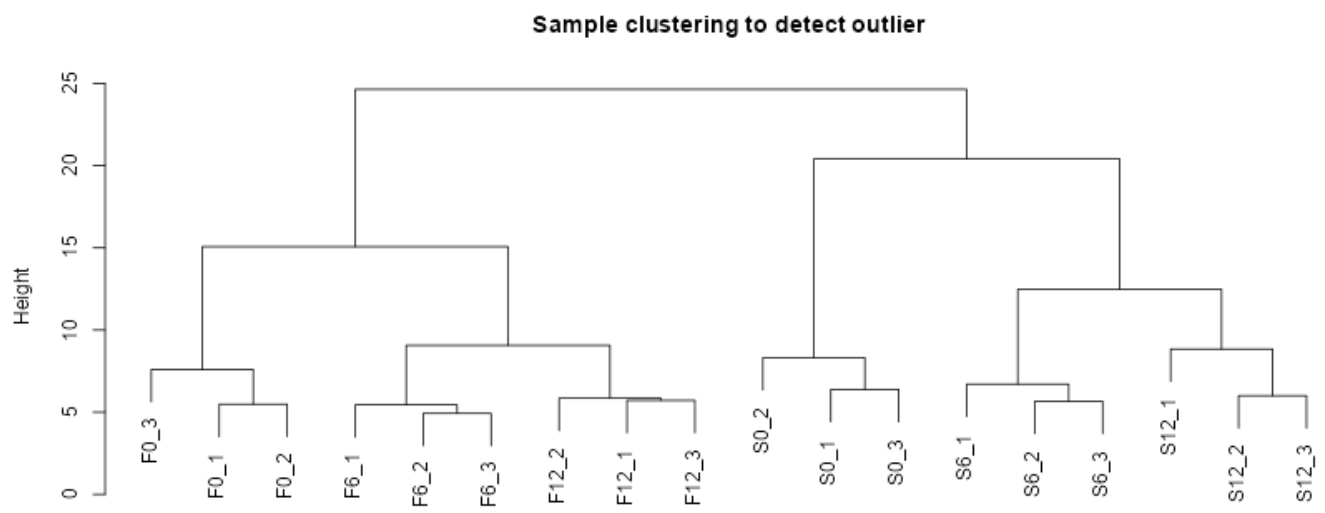

**Fig.S6** Phylogenetic tree diagram of metabolites from 18 samples.

Supplement: Supplementary file 6 — Supplementary Material 6. [file 12864_2024_11151_MOESM6_ESM.pdf]

A

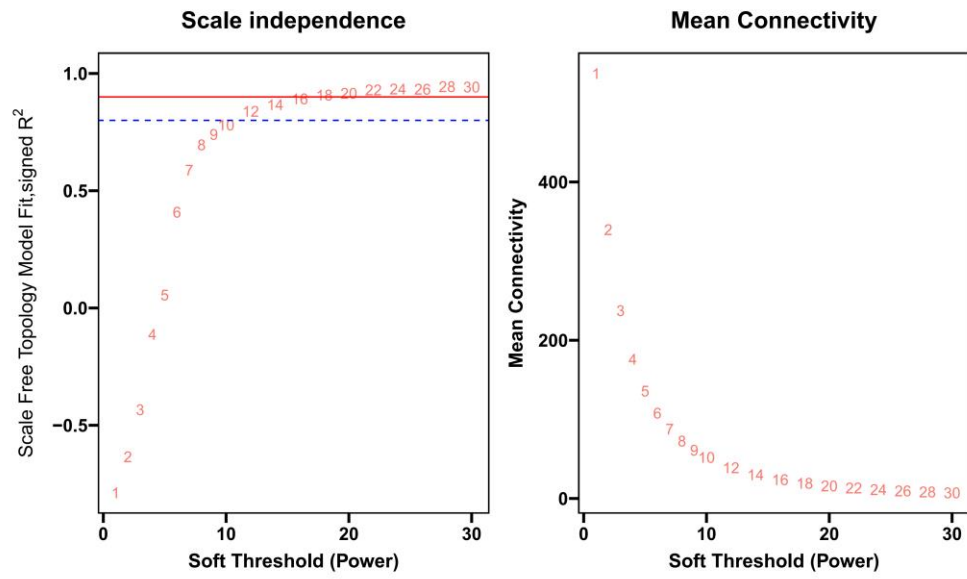

B

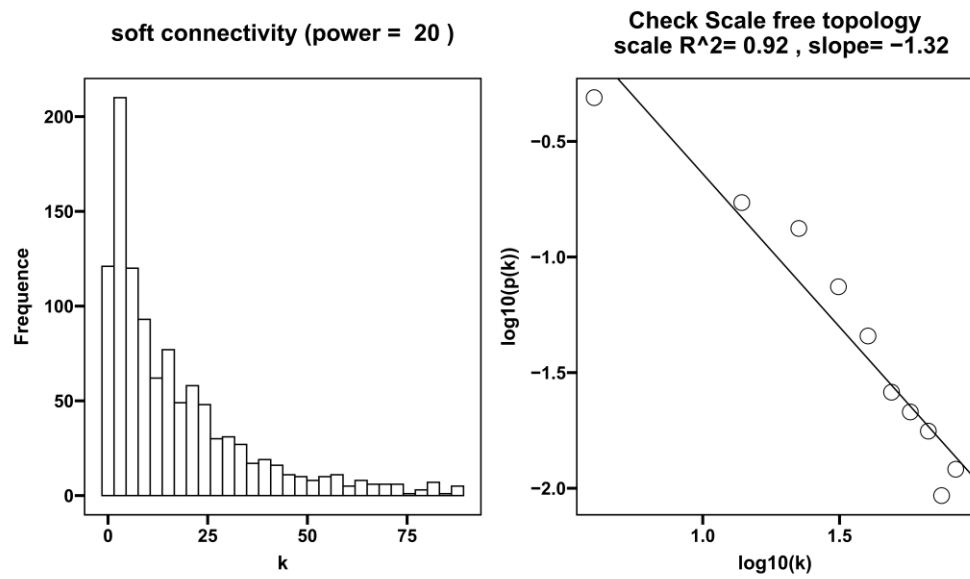

Fig.S7 (A) Network topology diagram of soft threshold power. (B) Check the scale-free topology map.

Supplement: Supplementary file 7 — Supplementary Material 7. [file 12864_2024_11151_MOESM7_ESM.pdf]

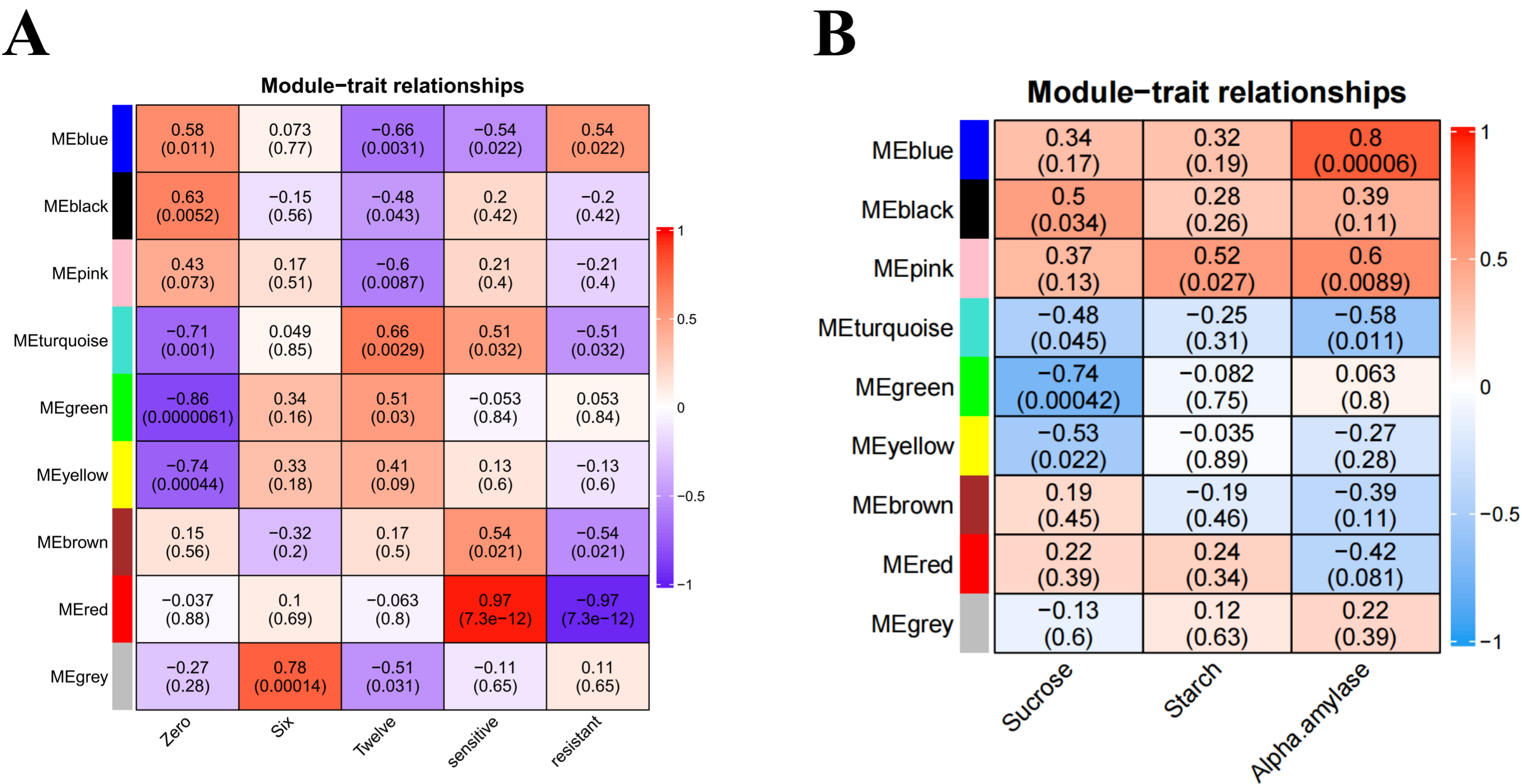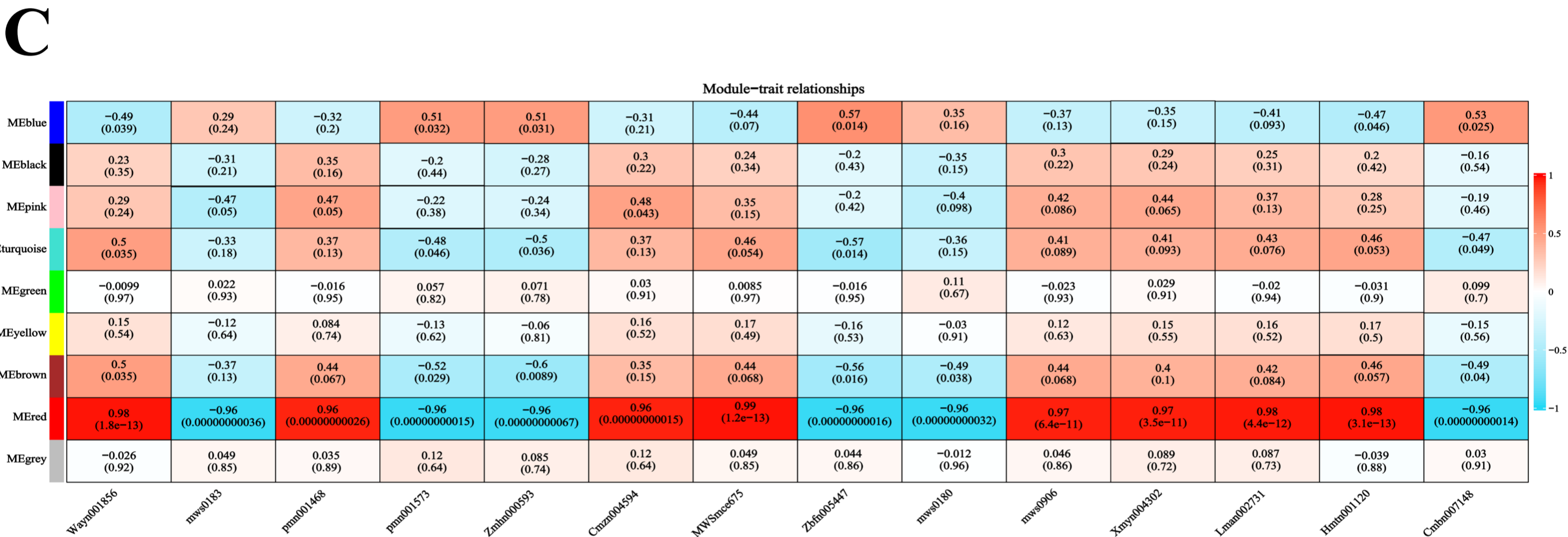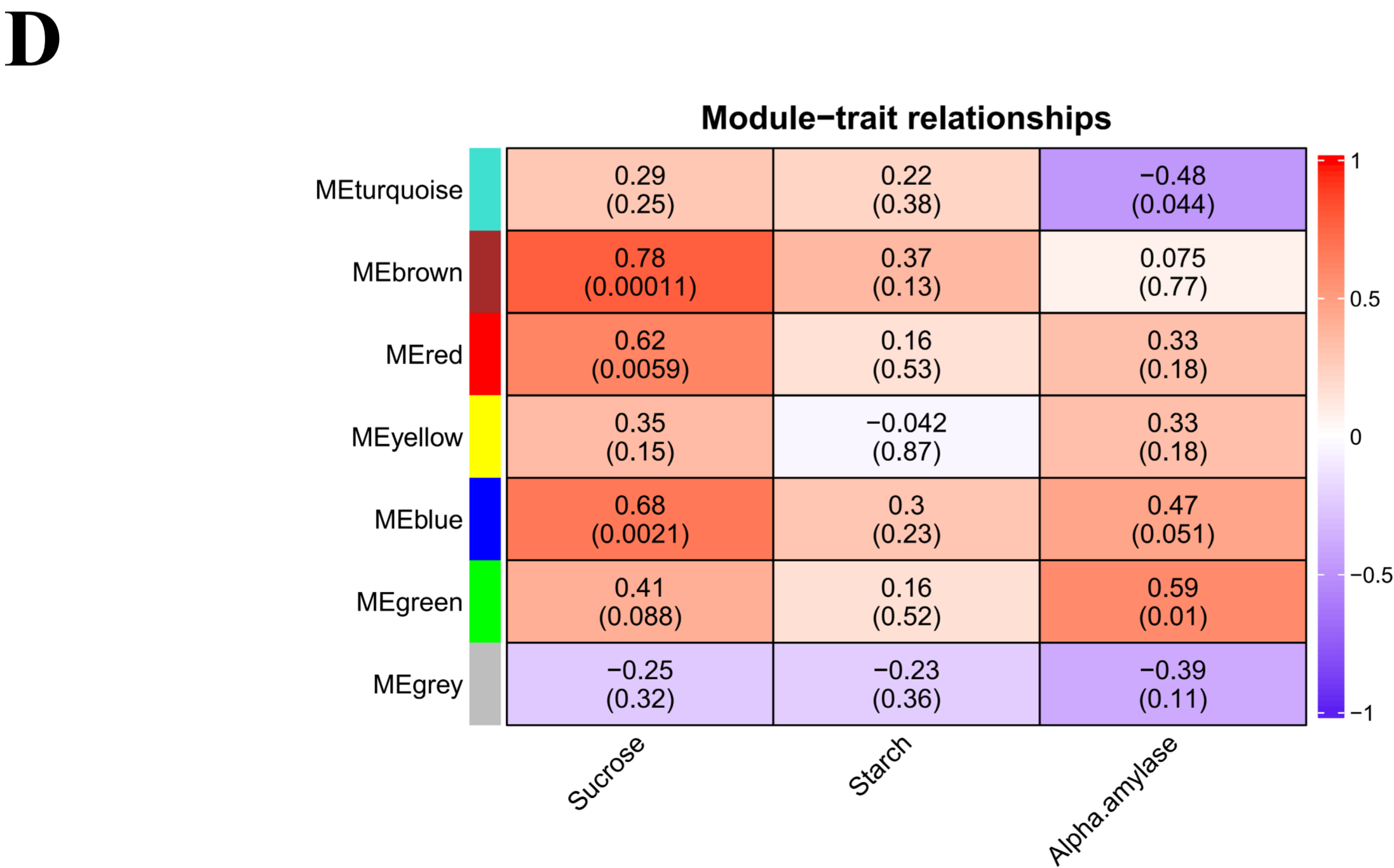

Supplement: Supplementary file 8 — Supplementary Material 8. [file 12864_2024_11151_MOESM8_ESM.pdf]

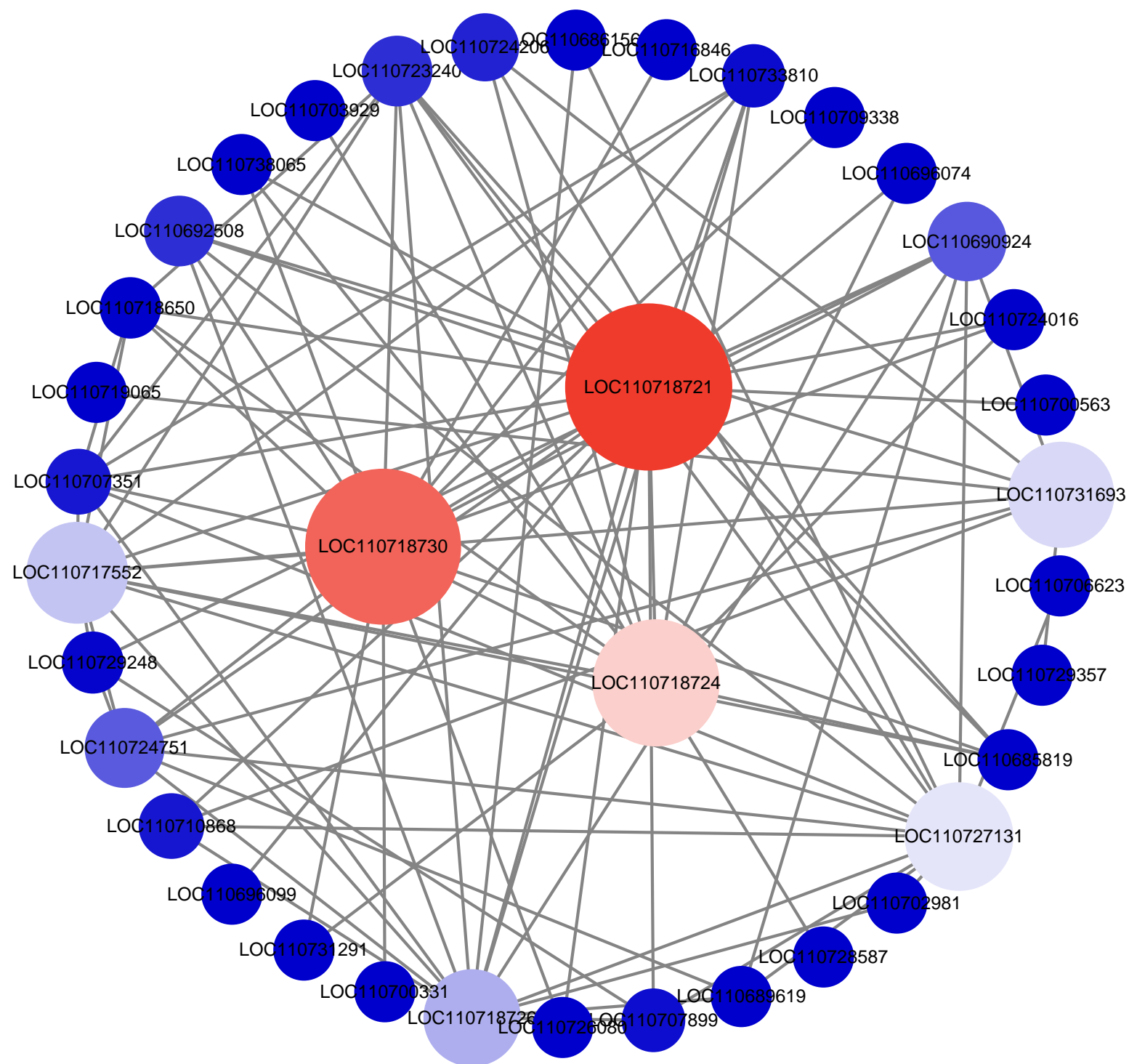

Supplement: Supplementary file 9 — Supplementary Material 9. [file 12864_2024_11151_MOESM9_ESM.pdf]

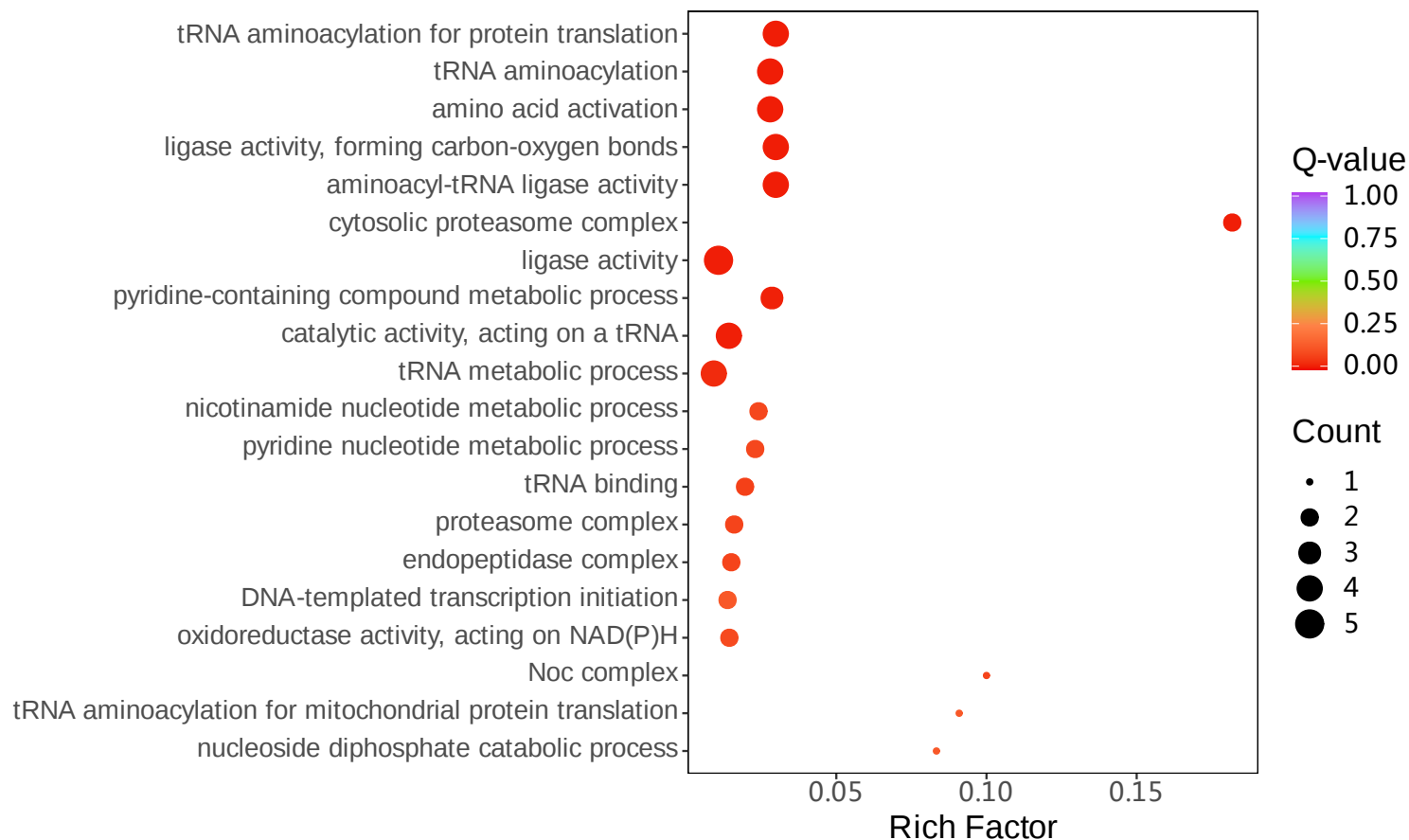

**Fig.S11** Go enrichment analysis.

Supplement: Supplementary file 11 — Supplementary Material 11. [file 12864_2024_11151_MOESM11_ESM.pdf]

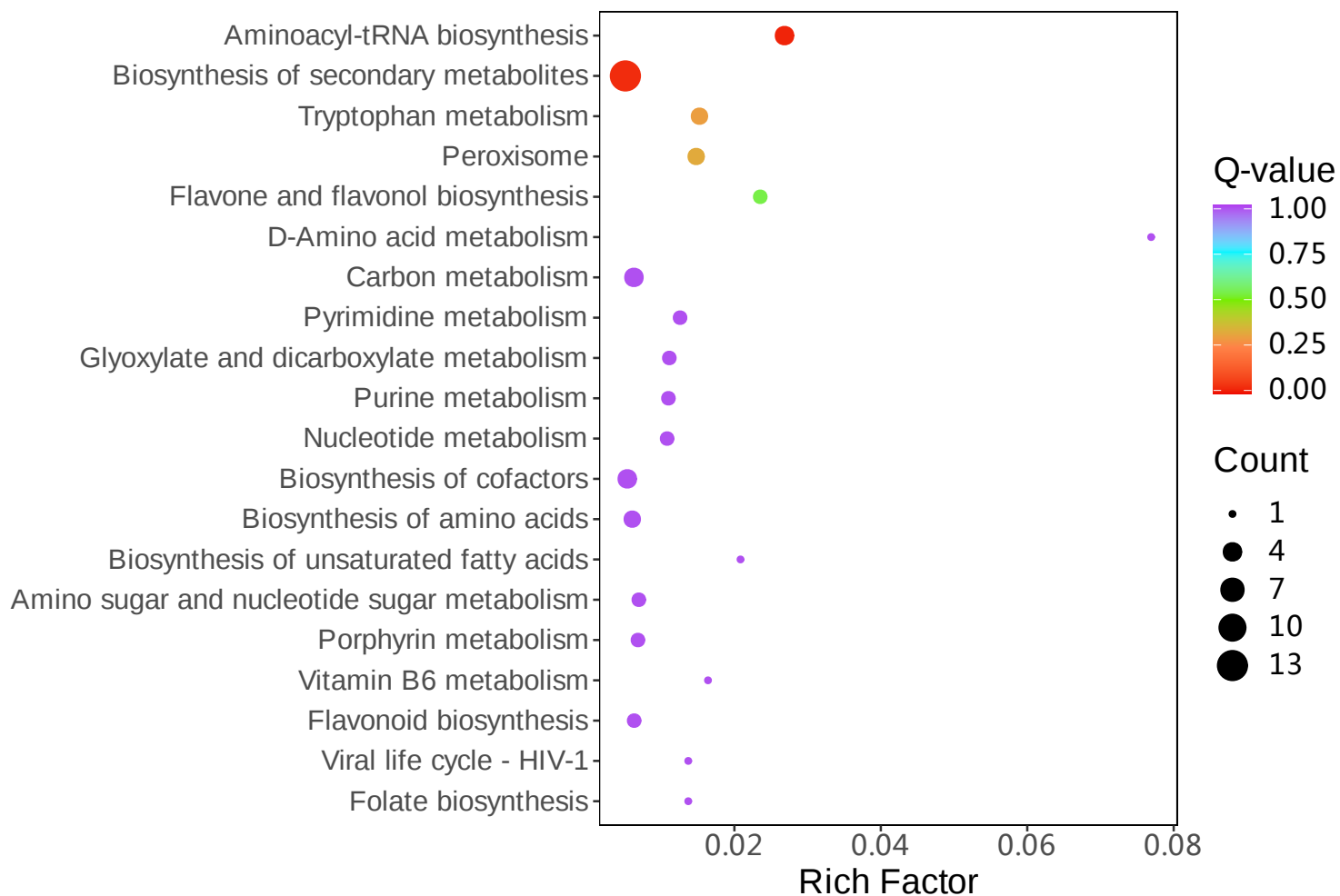

**Fig.S12** KEGG enrichment analysis

Supplement: Supplementary file 12 — Supplementary Material 12. [file 12864_2024_11151_MOESM12_ESM.pdf]
