# Supplementary material for: Comparative transcriptomes and WGCNA reveal hub genes for spike germination in different quinoa lines
Source: BMC Genomics. 2024 Dec 20;25:1231. doi: 10.1186/s12864-024-11151-y (PMC11662621; doi:10.1186/s12864-024-11151-y)
Supplement: Supplementary file 2 — Supplementary Material 2. [file 12864_2024_11151_MOESM2_ESM.pdf]

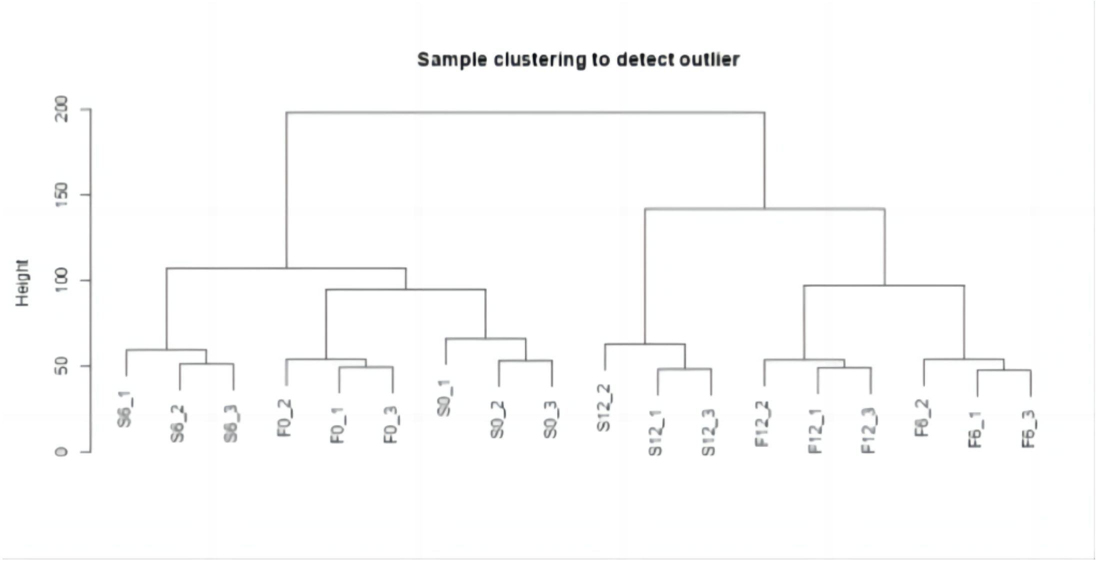

Fig.S2 Phylogenetic dendrogram of all 18 samples. The horizontal coordinates represent sample clustering, and one column represents one sample. The Clustering is based on the similarity of gene expression between samples. The closer the gene expression between samples is, the closer they are to each other (F represents red quinoa (Dianli-222), S represents red quinoa (Dianli-654); F0/S0 represents humidity treatment for 0 hours; F6/S6 represents humidity treatment for 6 hours; F12/S12 represents humidity treatment for 12 hours)
