# Supplementary material for: Comparative transcriptomes and WGCNA reveal hub genes for spike germination in different quinoa lines
Source: BMC Genomics. 2024 Dec 20;25:1231. doi: 10.1186/s12864-024-11151-y (PMC11662621; doi:10.1186/s12864-024-11151-y)
Supplement: Supplementary file 3 — Supplementary Material 3. [file 12864_2024_11151_MOESM3_ESM.pdf]

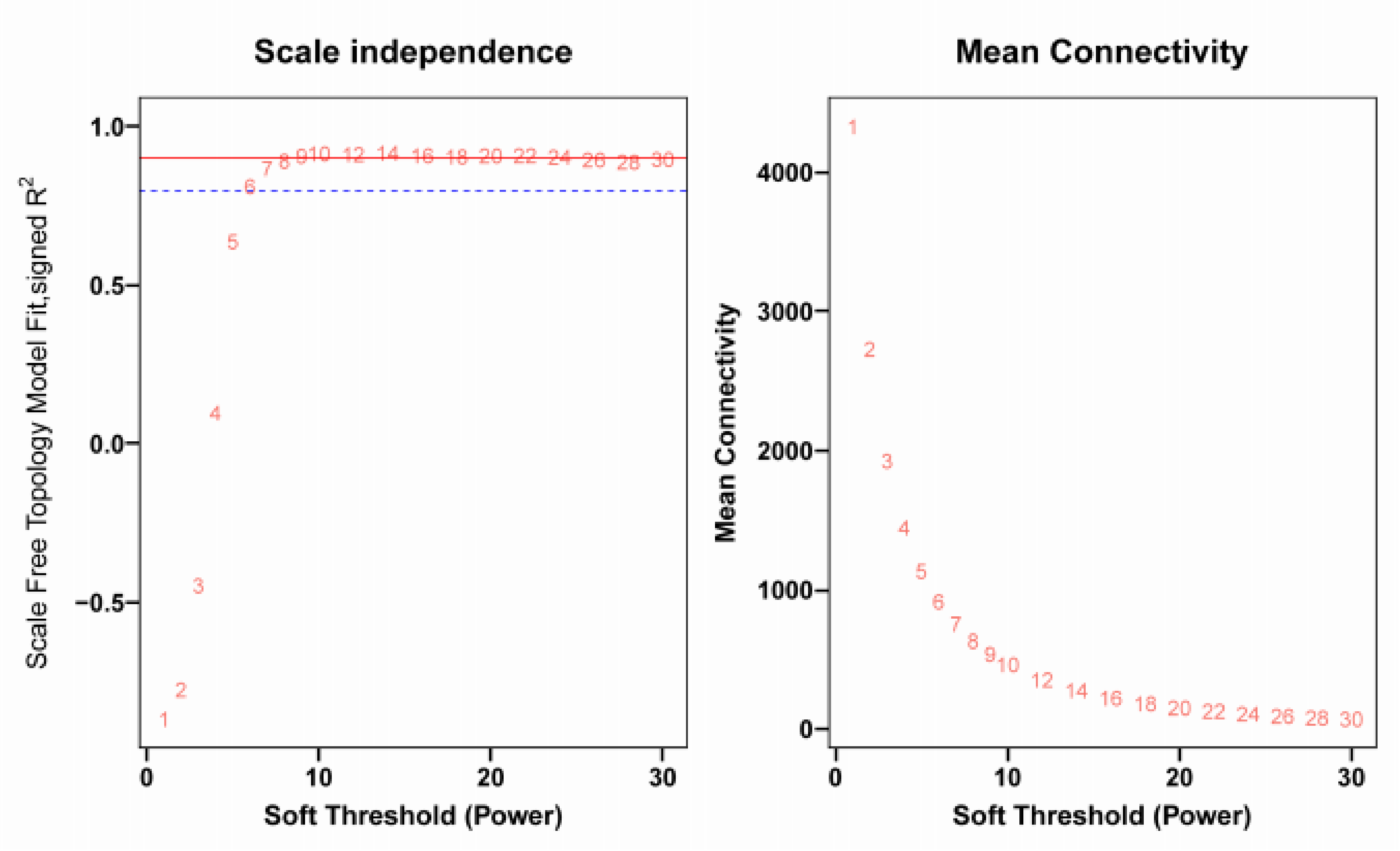

Fig.S3 Network topology for different soft-threshold powers for quinoa spike germination. The x-axis represents the weight parameter β. The vertical axis of the left panel represents the square of the correlation coefficients in the corresponding network, and the vertical axis of the right panel represents the average of the neighbor-joining functions of all genes in the corresponding gene module.
