# Supplementary material for: Comparative transcriptomes and WGCNA reveal hub genes for spike germination in different quinoa lines
Source: BMC Genomics. 2024 Dec 20;25:1231. doi: 10.1186/s12864-024-11151-y (PMC11662621; doi:10.1186/s12864-024-11151-y)
Supplement: Supplementary file 10 — Supplementary Material 10. [file 12864_2024_11151_MOESM10_ESM.pdf]

A

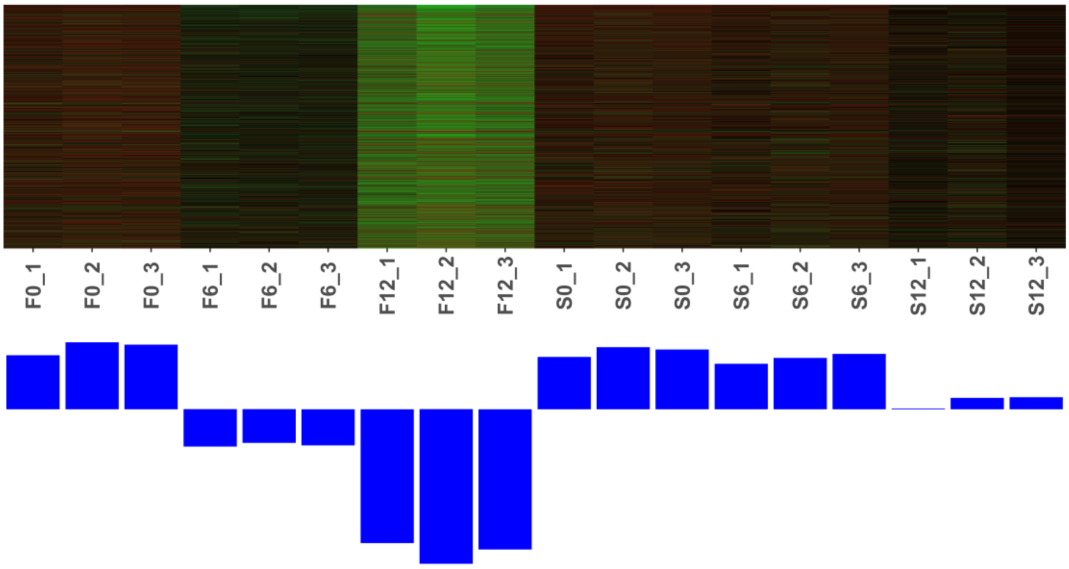

B

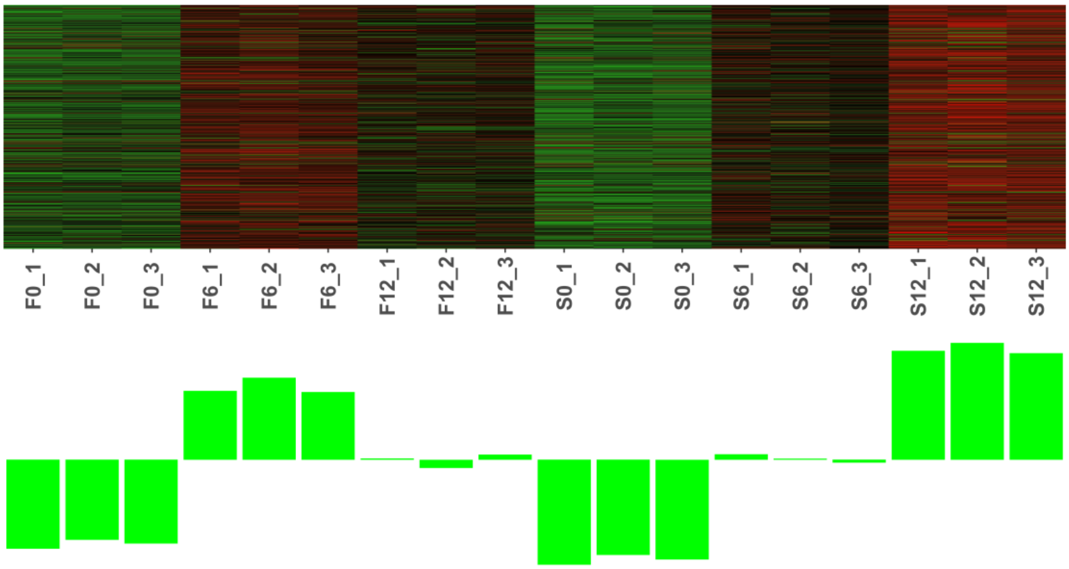

Fig.S10 Sample expression heat map of coexpression module. The figure above shows the eigenvalues of the modules in different samples, with red indicating up-modulation and green indicating down-modulation.
